# A Network of Topographic Maps in Human Association Cortex Hierarchically Transforms Visual Timing-Selective Responses

**DOI:** 10.1016/j.cub.2020.01.090

**Published:** 2020-04-20

**Authors:** Ben M. Harvey, Serge O. Dumoulin, Alessio Fracasso, Jacob M. Paul

**Affiliations:** 1 Experimental Psychology, Helmholtz Institute, Utrecht University, Heidelberglaan 1, 3584 CS Utrecht, the Netherlands; 2 Spinoza Center for Neuroimaging, Meibergdreef 75, 1105 BK Amsterdam, the Netherlands; 3 Experimental and Applied Psychology, VU University, Van der Boechorststraat 7, 1081 BT Amsterdam, the Netherlands; 4 Institute of Neuroscience and Psychology, University of Glasgow, 62 Hillhead Street, Glasgow G12 8QB, United Kingdom

**Keywords:** vision, timing, numerosity, topographic maps, hierarchy, human, brain, functional magnetic resonance imaging, fMRI, population receptive field, pRF

## Abstract

Accurately timing sub-second sensory events is crucial when perceiving our dynamic world. This ability allows complex human behaviors that require timing-dependent multisensory integration and action planning. Such behaviors include perception and performance of speech, music, driving, and many sports. How are responses to sensory event timing processed for multisensory integration and action planning? We measured responses to viewing systematically changing visual event timing using ultra-high-field fMRI. We analyzed these responses with neural population response models selective for event duration and frequency, following behavioral, computational, and macaque action planning results and comparisons to alternative models. We found systematic local changes in timing preferences (recently described in supplementary motor area) in an extensive network of topographic timing maps, mirroring sensory cortices and other quantity processing networks. These timing maps were partially left lateralized and widely spread, from occipital visual areas through parietal multisensory areas to frontal action planning areas. Responses to event duration and frequency were closely linked. As in sensory cortical maps, response precision varied systematically with timing preferences, and timing selectivity systematically varied between maps. Progressing from posterior to anterior maps, responses to multiple events were increasingly integrated, response selectivity narrowed, and responses focused increasingly on the middle of the presented timing range. These timing maps largely overlap with numerosity and visual field map networks. In both visual timing map and visual field map networks, selective responses and topographic map organization may facilitate hierarchical transformations by allowing neural populations to interact over minimal distances.

## Highlights

• Many brain areas show neural responses to specific ranges of visual event timing
• Timing preferences change gradually in these areas, forming topographic timing maps
• Neural response properties hierarchically transform from visual to premotor areas
• Timing, numerosity, and visual field map networks are distinct but largely overlap

## Teaser

Harvey et al. use advanced fMRI to reveal selective neural responses to specific ranges of visual event timing in a widespread network of topographically organized human brain areas. Timing representations are hierarchically transformed from visual to motor planning areas. These timing maps are distinct from numerosity maps but largely overlap.

## Introduction

Precisely quantifying sub-second sensory event timing is vital to understanding and interacting with events in our dynamic world. This is required for the perception and performance of speech, music, driving, and many sports: fast, complex behaviors that are unique to humans. Humans can perceive sub-second event durations and frequencies, compare these across senses, and synchronize motor actions to them. Recent fMRI studies have demonstrated responses in human early visual areas that monotonically change with visual event timing, increasing with event frequency and duration [1, 2]. For motor planning, macaque neurophysiological measurements demonstrate responses to motor event frequency in the macaque supplementary motor area, following both tuned and monotonic functions [3, 4]. But how are responses to visual event timing from early visual areas processed and transformed to allow multisensory integration and action planning to follow sensory timing?

Evidence from sensory timing perception [5] and computational modeling [6] suggests neural responses selective for specific sensory event durations or frequencies [7]. We recently demonstrated that selective responses to another sensory quantity [8], visual numerosity [9, 10], form an extensive fronto-parietal network of topographic maps [11, 12]. In this network, neural numerosity preferences change gradually across the cortical surface, with maps found in areas involved in visual object processing, multisensory integration, and action planning. These numerosity maps also largely overlap with responses to another quantity, object size [13], and with visual field maps. We therefore hypothesized that human cortical neural populations may exhibit selective responses to visual event timing in a network of topographically organized areas. One such topographic timing map was recently described at the motor planning stage, in human supplementary motor area, during a duration comparison task [14]. However, it is unclear whether other areas show similar responses, whether other areas show similar organization, whether the precision of timing selectivity varies systematically within or between maps, whether event frequency is also encoded in these timing responses [6], or whether timing maps are located together with responses to other quantities. We further hypothesized that the timing selectivity may be progressively transformed from extrastriate visual areas through multisensory integration areas to motor planning areas to optimize timing representations for different cognitive functions. Finally, we hypothesized that these timing responses may overlap with responses to other quantities and visual space.

**Figure 1:**
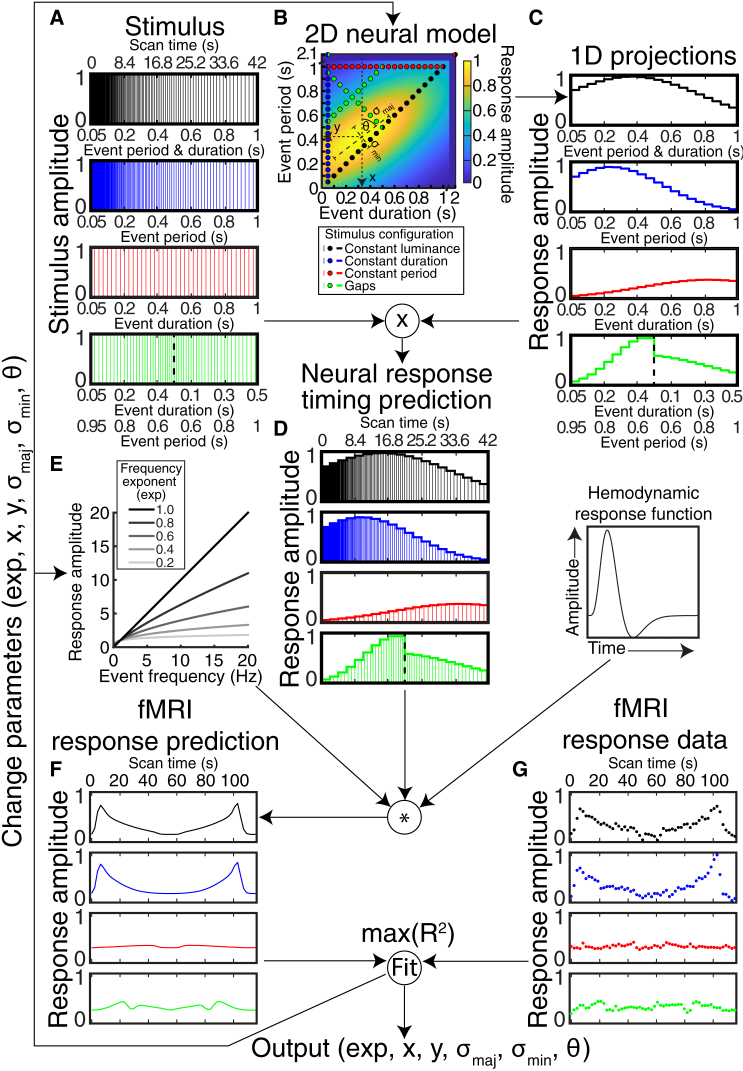
Deriving Timing Selectivity Parameters from fMRI Responses to Temporally Varying Stimuli (A) Stimulus time course, showing event duration and/or period (x axis) at event offsets (lines). (B) Stimulus states and response function, showing presented event durations and periods (dots) on one candidate response function (background image). (C) The response function’s amplitude at the event timings shown in the stimulus time course. (D) Predicted neural response amplitude at each presented event’s offset, proportional to the amount of color under the curve. (E) Compressive exponent to capture sub-additive increases in neural response amplitude with increasing event frequency. (F) fMRI response time course predicted by the neural response amplitude (D) scaled by the compressive exponent (E) and convolved with the hemodynamic response function. Note fMRI responses are shown for increasing and decreasing stimulus progressions, whereas previous panels show increasing progressions only. (G) Example recording site’s measured fMRI response time course. The compressive exponent (exp) and the following response function parameters were fit to maximize the correlation (R^2^) between predicted and measured fMRI response time courses: preferred duration (x), preferred period (y), response function extent along its major (σ_maj_) and minor (σ_min_) axes, and major axis orientation (θ). See also Figure S1 and Video S1.

We acquired ultra-high-field (7T) fMRI data while showing repetitive visual events (a circle repeatedly appearing and disappearing) that gradually varied in duration and/or frequency (Figure 1A; Video S1). Gradual timing changes reveal timing selectivity at fMRI’s slow timescale. Human timing perception studies typically vary the duration of single events [5], whereas animal timing neurophysiological studies typically vary the interval between stimuli or actions [3]. Unifying these approaches, during each scan, we systematically varied both event duration and event period (the time between repeating event onsets; i.e., 1/frequency) ranging from 50 ms to 1 s in steps of 50 ms. We express event frequency in terms of period because both period and duration are measured in seconds, allowing straightforward comparison. Event duration co-varies with either mean display luminance (if events begin at regular intervals) or event period (if each event is immediately followed by another). Controlling for these correlated properties, we characterize four stimulus configurations (Figure 1A; Video S1). The “constant luminance” configuration matched duration and period so an event was always ongoing. The “constant duration” configuration presented 50-ms events with varying period. The “constant period” configuration presented events with varying duration and 1,000-ms period. Finally, the “gaps” configuration varied both duration and period to sample timings absent in other configurations. Subjects made no duration or period judgments but reported when white circles were presented rather than black (performance > 80%). This happened pseudo-randomly, equally frequently for all timings.

Video S1. Event Timing Stimuli in Each Stimulus Configuration, Related to Figure 1 Text and distance markers were not shown, and stimulus configuration blocks were shown in a pseudo-random order differing between scan runs.

## Results

### Timing-Selective Responses

**Figure 2:**
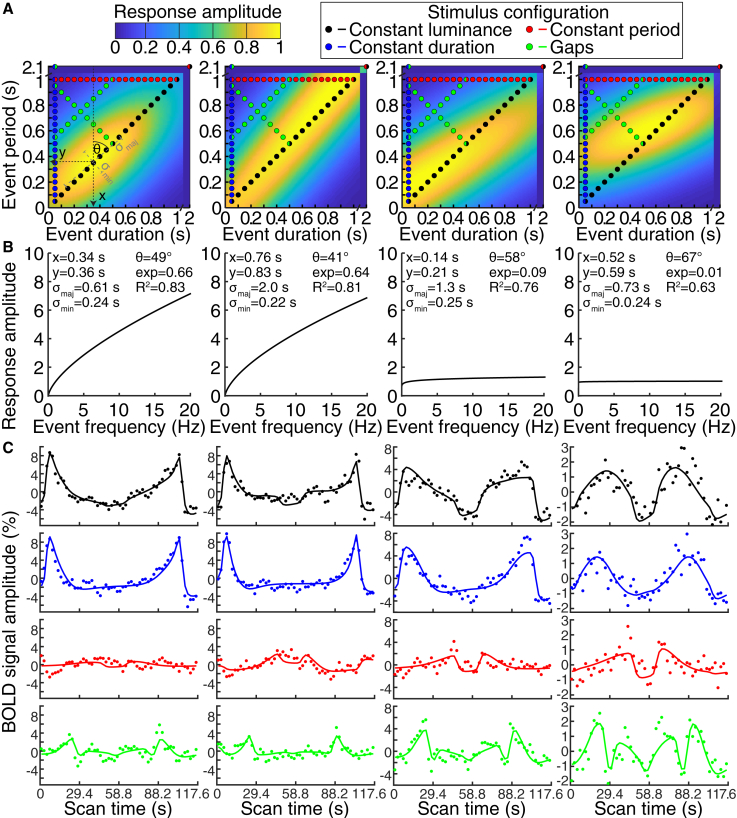
Different Example Recording Site Responses Captured by Different Timing Response Parameters (A) In each recording site (columns), we fit the timing response function (background) that best predicts responses to all stimulus configurations’ event timings (colored circles). (B) Responses accumulate sub-additively with event frequency, summarized by fitting a compressive exponent. (C) Each stimulus configuration samples its event timings in both directions. At every event offset, we evaluate the candidate response function’s amplitude, scale this by the frequency exponent, and convolve the result with a hemodynamic response function (see Figure 1). This predicts an fMRI response time course (colored lines), which we correlate to the measured response (colored dots). For clarity, responses shown here are from recording sites with particularly good model fits. Response function parameters preferred duration (x), preferred period (y), major (σ_maj_) and minor (σ_min_) axis extents, major axis orientation (θ), and compressive exponent (exp) are fit to maximize this correlation (R^2^). See also Video S1.

We summarized the responses of each recording site (voxel) to all four stimulus configurations using one neural response model (see STAR Methods; Figure 1) [15]. We tested the predictions of several candidate neural response functions (Figure S1) that each predicted how each recording site’s response amplitude changed with stimulus event timing. At every event offset, we evaluate the candidate response function’s amplitude and scale this by the frequency exponent (Figures 1A and 1E). This predicts the neural response amplitude time course of this candidate response function to our stimulus sequence’s event offsets. Convolving this with a hemodynamic response function predicts an fMRI response time course, which we correlate to the measured response (Figures 1F and 1G). We repeat this for a large set of parameters of the response function and find those that produce the prediction best correlated with each recording site’s response time course. This determined the timing response selectivity (tuning) parameters (Figures 2A and 2B) that predict the fMRI response time course best correlated to the recording site’s observed responses (Figure 2C).

We tested several potential parametric response models (Figure S1). Comparing potential model fits on cross-validated data let us distinguish between these models’ performance in predicting observed responses despite differences in model complexity. Tested models included selectivity for temporal frequency; event duration and period; event period and occupancy (proportion of that period the event filled; i.e., period/duration); and event duration and inter-event interval (time from event offset to the next event onset; i.e., period−duration). We also compared logarithmic and linear response functions, and monotonic amplitude increases with occupancy (i.e., sustained neural response component) and/or event onset frequency (i.e., transient components) [1, 2].

A 2-dimensional anisotropic Gaussian function selective for duration and period, with a compressive exponent on event frequency, best predicted the observed responses (t test of map mean response variance explained against next best model, paired across maps: p < 10^−10^, t = 8.3, n = 304, mean 31.7% versus 30.8% variance explained) (Figures S1J and S1K). Even comparing each subject’s global mean response variance (rather than each map’s) between models, this model outperformed all others (against the next best model: p = 0.016, t = 3.17, n = 8), suggesting the best fitting model generalized across our population of subjects. This best fitting model’s response function had five parameters (Figures 1B, 2A, and 2B). The first two were the preferred duration and period, yielding the largest response per event. Each recording site responded to a range of timings, summarized by the function’s extent along its major and minor axes, and the major axis’s angulation (third, fourth, and fifth parameters). A sixth parameter, compressive exponent, captured sub-additive increases in response amplitude with increasing event frequency (Figures 1E and 2B) [2]: at an exponent of one, response amplitudes would be 10 times larger for 10 events per s than 1 event per s, whereas at an exponent of zero these response amplitudes would be identical.

Temporal frequency-selective responses are common in early visual cortex. However, this model’s predictions captured the measured responses poorly. Notably, our events have immediate onsets and offsets with broad temporal frequency power distributions, whereas narrow temporal frequency stimuli (such as sinusoidal luminance modulations) lack discrete events with meaningful durations. Furthermore, power spectra do not distinguish between an event’s duration and its inter-event interval, although their phase differs. Therefore, although a 50-ms event with a 950-ms inter-event interval produces a different response to a 950-ms event with a 50-ms inter-event interval, their power spectra are identical.

### Topographic Timing Maps

**Figure 3:**
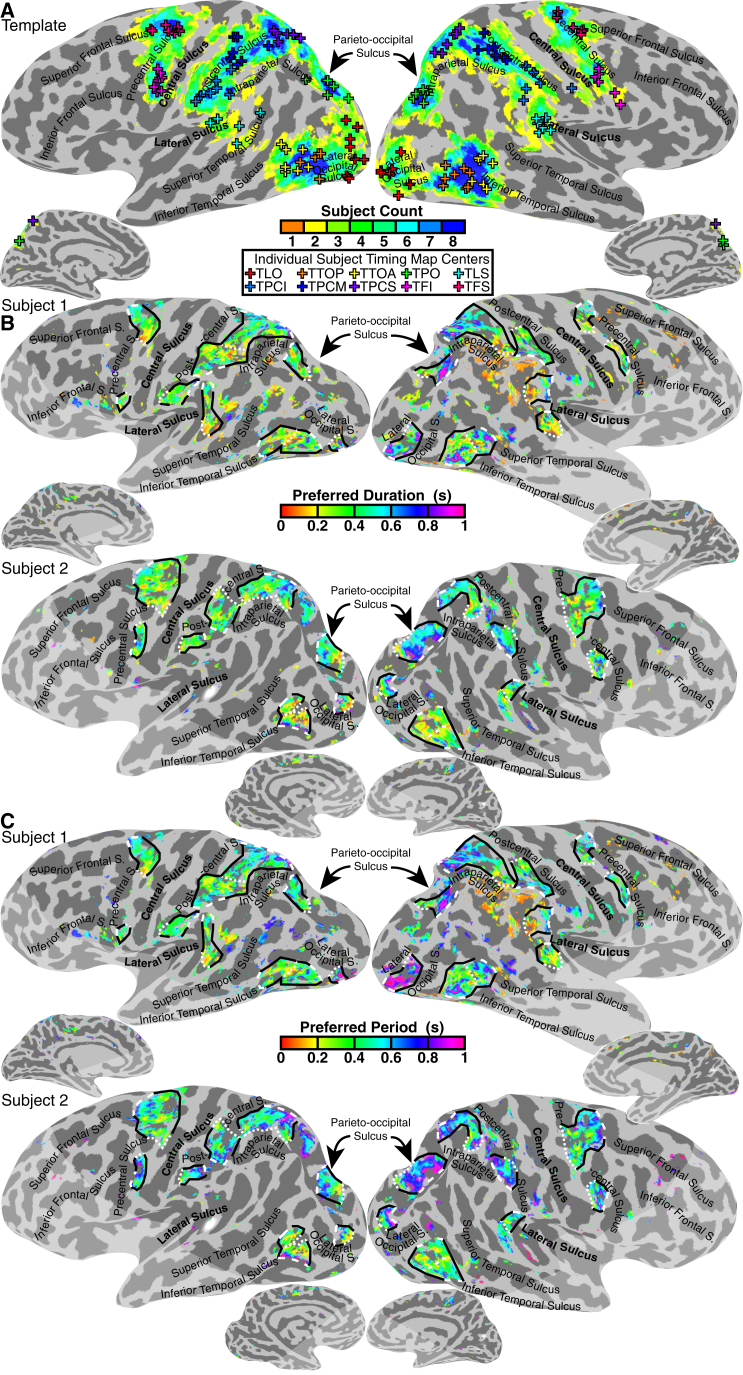
Cortical Locations of Timing Maps and Their Progressions of Preferred Duration and Period (A) Timing map locations transformed onto the N27 (Talairach) template’s cortical surface anatomy (grays; major sulci are labeled in black). Colors show the number of subjects whose maps overlap with each surface location, whereas crosses show the transformed locations of individual subjects’ map centers. See also Table S1 and Figures S2 and S3. (B) Projecting each recording site’s preferred duration (colors; for recording sites with over 10% response variance explained by the response model) onto the subject’s cortical surface anatomy reveals ten maps, each containing a range of timing-selective responses (outlined with black and white lines: fine dashed white lines connect map border sites with the lowest duration preferences, and coarse dashed white lines connect map border sites with the highest duration preferences). See also Figure S2. (C) Preferred period of the same recording sites. See also Figure S3.

We found ten maps on each hemisphere’s lateral surface (with none on the medial surface) where these models captured response variance best, consistently positioned relative to major sulci (Figure 3A). These form the basis of our regions of interest. We named these maps by their anatomical locations, preceded by “T” for “timing,” following naming conventions for visual field maps [16] and numerosity maps [11]. Table S1 gives their Montreal Neurological Institute coordinates. Moving posterior/inferior to anterior/superior, TLO (timing lateral occipital) lay in the lateral occipital sulcus. TTOP (timing temporo-occipital posterior) and TTOA (timing temporo-occipital anterior) lay on the inferior lateral boundary of the temporal and occipital lobes, in the posterior inferior temporal sulcus, consistent with the human MT+ (or TO [temporal-occipital]) visual field maps’ location [17, 18]. TPO (timing parieto-occipital) lay superior to the parieto-occipital sulcus, medial to the posterior intraparietal sulcus (IPS). TLS (timing lateral sulcus) lay in the posterior lateral sulcus. TPCI (timing postcentral inferior) lay in the inferior postcentral sulcus. TPCM (timing postcentral medial) lay in the middle of the postcentral sulcus near its junction with the IPS. TPCS (timing postcentral superior) lay immediately posterior to the superior postcentral sulcus and medial to the anterior IPS. TFI (timing frontal inferior) and TFS (timing frontal superior) lay in the premotor cortex, at the intersection of the precentral sulcus with the inferior and superior frontal sulci, respectively.

**Figure 4:**
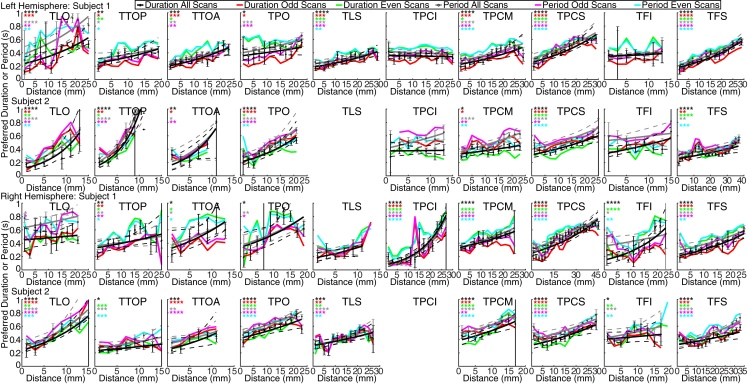
Quantifying Topographic Progressions of Timing Preferences across the Cortical Surface Progressing across each map in Figures 3B and 3C (from fine to coarse dashed white lines) reveals gradual topographic progressions of preferred duration and period (fit lines and points) that are reproduced across repeated measures (colored lines). See also Figure S4. ^∗^p < 0.05, ^∗∗^p < 0.01, ^∗∗∗^p < 0.001, ^∗∗∗∗^p < 0.0001. Points show bin mean values; error bars show SEs.

We identified maps in 155 of the 160 locations tested (10 locations × 8 subjects × 2 hemispheres). Projecting each recording site’s preferred duration or period (Figures 3B and 3C) onto the cortical surface revealed that both progressed gradually across the cortical surface, forming topographic timing maps. We quantified this progression by sorting recording sites within each map by their distance from the map borders with the lowest and highest preferred durations (white lines in Figures 3B and 3C). Recording sites’ preferred durations were significantly correlated with cortical distance (at p < 0.05) in 101 of 155 maps (65%), and preferred period was correlated with cortical distance in 89 (57%) (Figures 4 and S4). In split halves of the dataset, preferred duration and cortical distance were significantly correlated in 149 of 310 (2 × 155) map measurements (48%), and preferred period and cortical distance were significantly correlated in 128 (41%). Preferred duration estimates from odd and even runs were significantly correlated in 55 of 155 maps (35%), whereas preferred period estimates were correlated in 65 (42%). This repeatability over independent measures reached significance more often in the superior (and larger) maps (TPO, TPCM, TCPS, TFS), where 36 of 64 maps (56%) showed correlated duration preferences and 38 (59%) showed correlated period preferences between odd and even runs (Figure S4). As such, we are more confident of the repeatable topographic organization of the larger maps than of the smaller maps we describe. Such gradual progressions of preferred timing group similarly responding neural populations [19, 20], allowing us to characterize neural response selectivity at fMRI’s limited spatial resolution.

**Figure 5:**
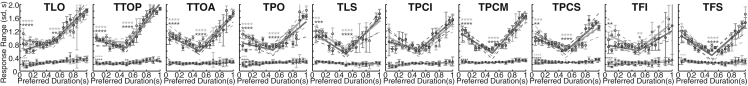
Changes in Response Function Extent within Timing Maps In data grouped across subjects, response function extent systematically increased with preferred duration in posterior maps (and along the minor axis) and with distance from the middle of the presented range in the major axis of anterior maps. Left and middle stars show the significance of increase with preferred duration and distance from the middle of the presented range, respectively. Large and small stars show the significance of major and minor extent increases, respectively. ^∗^p < 0.05, ^∗∗^p < 0.01, ^∗∗∗^p < 0.001, ^∗∗∗∗^p < 0.0001. Points show bin mean values; error bars show SEs.

Within each map, individual recording sites’ response function extents varied systematically with preferred duration in two distinct ways (Figure 5). First, the response function’s major and minor extents increased significantly with preferred duration, except in right TFS (for the major extent) and left TTOA, and bilateral TLS and left TFI (for the minor extent). Second, the major extent increased significantly as the preferred duration moved away from the middle of the presented range. So, the most posterior timing map responded to brief timings most specifically, whereas the others focused on the middle of the presented range, suggesting range-dependent timing selectivity.

### Differences between Maps

**Figure 6:**
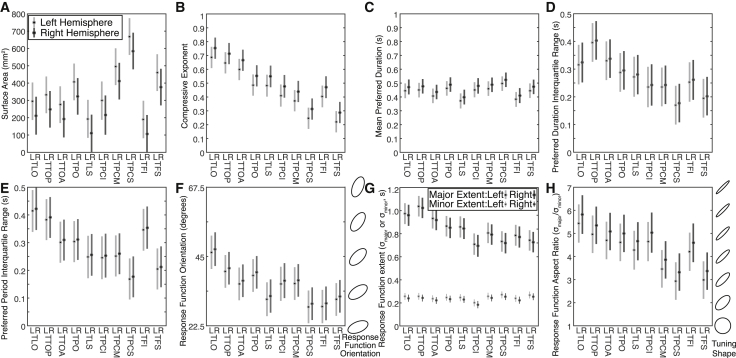
Differences between Timing Maps (A) Left-hemisphere maps have larger surface areas than right-hemisphere maps, and map surface areas increase from inferior to superior maps. (B) The compressive event frequency exponent decreases from posterior to anterior maps, from inferior to superior along the postcentral (TPCI-TPCS) and precentral (TFI-TFS) sulci, and in the left hemisphere. (C) The mean preferred duration of recording sites differed little between maps. (D and E) The interquartile range of preferred durations (D) and periods (E) of each map’s recording sites decreases from posterior to anterior. (F) Response function orientation becomes more horizontal from posterior to anterior. (G) The response function major extent (timing range to which individual recording sites respond) decreases from posterior to anterior. The minor extent does not change. (H) The mean response function aspect ratio (i.e., extent of response function elongation) therefore decreases from posterior to anterior. Points represent the population marginal mean of values across subjects. Error bars are 95% confidence intervals: separable error bars show significant differences at p < 0.05.

Map sizes (Figure 6A) and the goodness of response model fits both showed left lateralization of timing responses. Three-way ANOVAs (factors: hemisphere, map, and subject) reveal greater cortical surface areas (p = 0.0009, F(1,153) = 11.6) and better model fits (p = 0.0003, F(1,153) = 13.9) in left-hemisphere maps, a partial left lateralization of timing responsivity. Surface areas also differed between maps (p < 10^−10^, F(9, 145) = 13.8), primarily increasing from inferior to superior maps.

We captured sub-additive accumulation of response amplitude with increasing event frequency as a compressive exponential function (Figures 1E and 2B). A three-way ANOVA revealed this exponent is smaller (more sub-additive accumulation) in the left hemisphere (p = 0.0003, F(1,153) = 14.1) and differs between maps (p < 10^−10^, F(9, 145) = 31.9). It decreases from posterior to anterior (Figure 6B) and from inferior to superior up the postcentral and precentral sulci. So, responses to repeating events are hierarchically integrated through this timing network, resembling similar integration of visual space through the visual field map hierarchy [2, 21].

Recording sites within each map had a range of preferred durations and periods. The mean preferred duration across all maps was 452 ms (Figure 6C), slightly below the presented range’s center (500 ms), whereas the mean preferred period was 556 ms, slightly above. Mean preferred durations and periods were correlated across maps (p < 10^−10^, r = 0.64). Preferred durations of each map’s recording sites were also correlated (p < 0.05), with their preferred periods in 144 of 155 maps (93%). Therefore, duration and period selectivity co-varied within and between brain areas, and between subjects.

Two-way ANOVAs of interquartile ranges of preferred duration and periods within each map (factors: map and subject; no significant hemisphere effect) revealed these differed between maps (duration: p = 10^−7^, F(9,145) = 6.6; period: p = 9 × 10^−9^, F(9,145) = 7.4). Primarily, the range of both duration (Figure 6D) and period preferences (Figure 6E) decreased from the posterior (TLO, TTOP) to the anterior superior (TPCS, TFS) maps. So, timing selectivity was transformed along the hierarchy to focus increasingly on the middle of the presented range, again suggesting range-dependent timing selectivity.

The timing response function was consistently angulated so that the event duration yielding the maximum response depended on the event’s period (and vice versa) (Figure 6F), further linking duration and period selectivity. A two-way ANOVA revealed this orientation differed between maps (p = 10^−9^, F(9,145) = 8.2) progressing from diagonal (equal extent in duration and period) in the most posterior maps to more horizontal (broader extent in the duration than period) toward the anterior maps. So, the precision of period selectivity became finer than that of duration selectivity through the hierarchy.

A two-way ANOVA revealed differences between maps in mean major axis extent (p < 10^−10^, F(9, 145) = 11.2). Primarily, the response function shortened from posterior (TLO, TTOP, TTOA) to anterior maps (TPCI, TPCM and TPCS; TFI and TFS) (Figure 6G), demonstrating finer timing selectivity in the anterior timing map hierarchy. However, the minor axis extent did not differ between maps, so the response function’s elongation also decreased from posterior to anterior maps (Figure 6H) (map effect on response function aspect ratio: p = 10^−8^, F(9, 145) = 7.3).

### Relationships to Numerosity and Visual Field Maps

**Figure 7:**
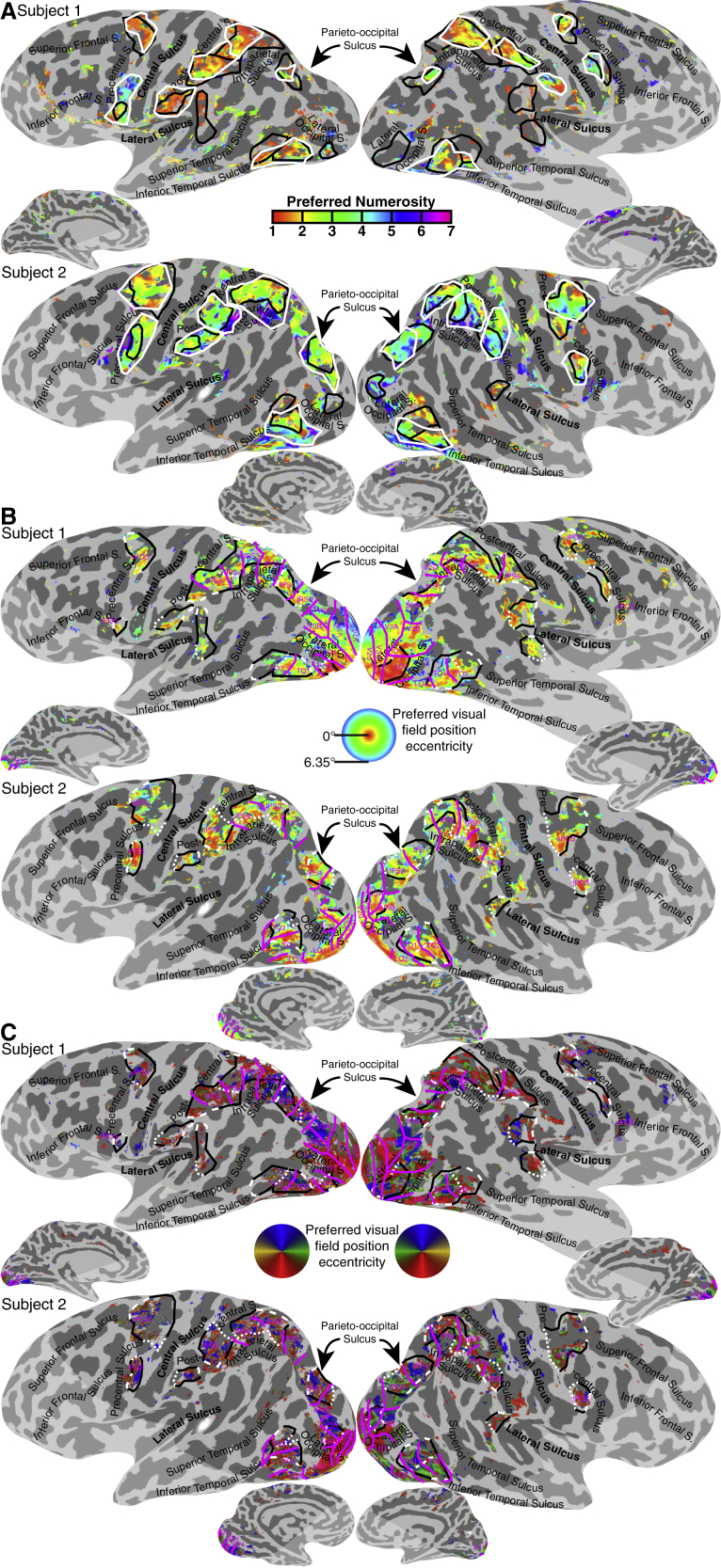
Numerosity and Visual Field Position Preferences (A) Numerosity preferences for recording sites with over 25% response variance explained by the response model. Numerosity maps (white outlines) overlap considerably with timing maps (black outlines). See also Figure S5. (B) Eccentricity preferences for recording sites with over 10% response variance explained by the response model. Timing map locations (black and white outlines) largely fall within the visual field map hierarchy. Visual field map borders are shown as magenta lines, and named in magenta text. See also Figure S6. (C) Polar angle preferences. See also Figure S7.

These timing maps largely overlapped with the subjects’ numerosity maps (Figure 7A). However, numerosity and timing networks appear to be distinct: there was no numerosity map near TLO or TLS; left-lateralized timing responses contrast with right-lateralized numerosity responses [11]; and TTO (timing) was consistently anterior to NTO (numerosity).

The visual field maps (Figures 7B and 7C) largely included the visual timing maps, unsurprising, as both are visually driven responses [11]. However, visual field maps and visual timing map borders did not coincide, their relative positions differed between subjects, and there were large areas of the visual field map network lacking timing selectivity. So, timing, numerosity, and visual position appear to produce distinct responses. Relationships between these (beyond similar locations and topographic organization) remain unclear, and further analyses will be necessary to quantitatively investigate these relationships.

## Discussion

Many studies describe timing-modulated responses in basal ganglia, hippocampus, and cerebellum. Here we focus on cortical responses. Recent fMRI results from Protopapa and colleagues show similar responses with topographic organization in human supplementary motor area (SMA) [14]. This appears to be at a different location from our TFS map, which lies in premotor cortex rather than SMA. Task differences may underlie this discrepancy. Protopapa et al.’s task relied on internally guided actions following duration comparisons, consistent with SMA’s role in internally generated motor plans [22, 23]. Macaque experiments [3, 4] showing frequency-selective responses in SMA similarly use internally guided rhythm continuation. Our subjects responded to stimulus color changes, an externally cued action consistent with premotor cortex’s role in externally cued motor planning.

Previous fMRI studies describe cortical activation near TLO, TFS, and TFI during visual duration comparison tasks [14, 24] and repetition suppression near TPCI and TLS by duration adaptation [25]. Areas near TPCM, TPCS, and right TFI allow visual event duration decoding, with right parietal decoding accuracy correlated to subjects’ behavioral timing discrimination performance [26]. Furthermore, parietal and frontal lesions can disrupt timing judgments, as can transcranial magnetic stimulation of frontal cortex, parietal cortex, and MT+ [24, 27]. These results may all reflect activity in the network we describe, and link it to timing perception. The responding locations suggest roles for timing-selective responses in visual perception, attention control, multisensory integration, and action planning. Hierarchical changes suggest sensory-motor transformations: from fast, broad-range sensory responses to slower motor responses following the presented range [28].

Macaque lateral intraparietal (LIP) neurons build to maximal firing rates at remembered, task-relevant visual event durations or periods [29, 30]. LIP’s human homolog is near TPCS and TPO, whereas macaque SMA’s is near TFS. However, human parietal and frontal lobes are greatly expanded compared to macaques. Establishing inter-species homologies is difficult here [31]. Human timing responses and analysis may be considerably more complex (as for numerosity [32]), potentially facilitating complex, timing-dependent behaviors.

Timing preferences do not cover the entire presented range, and tend to focus on the middle of the presented range moving anterior/superior in the hierarchy. TLO, TTOP, and TTOA have interquartile ranges of timing preferences between 0.3 s and 0.4 s. An even distribution of timing preferences between 0 and 1 s would give an interquartile range of 0.5, so here different timings are represented fairly evenly. By TCPS and TFS (the most anterior/superior maps), these interquartile ranges are around 0.2 s (50% of timing preferences in the narrow 0.4–0.6 s range). However, our recording sites group the responses of many nearby neurons [15, 33]. If this population of neurons contains all timing preferences equally (i.e., high scatter of timing preferences), this aggregate population will prefer the average, although the population of individual neurons may show a broad distribution. Despite this, we propose that this hierarchical narrowing of the timing preference range would likely be observed in populations of individual neurons because an increase in scatter of timing preferences should be accompanied by a larger population response function, and we observe smaller response function extents up the hierarchy. These considerations also suggest that the population response function extents we measure are larger than those of individual neurons [15].

Posterior maps show response function major extents increasing with duration, which may underlie timing perception’s scalar property (Weber’s law): decreased perceptual precision for longer timings [34]. Likewise, the anterior maps’ decrease in response function extent at the middle of the presented range may underlie timing perception’s regression to the mean (central tendency): bias of perceptual estimates toward the mean of the presented range [35]. Typically interpreted in a Bayesian framework, this gives a prior distribution of expected event timings with the highest precision at the middle of the expected range. We speculate that more anterior areas may show this effect more strongly to guide action planning most accurately for the most likely sensory event timings.

Although all timing maps are consistently positioned across subjects, only TPO, TPCM, TPCS, and TFS (i.e., the larger maps) are consistently oriented. This variability resembles that of many numerosity maps [11] and also fronto-parietal visual field maps [36, 37]. This variability of anterior topographic map orientations may arise because these maps are not constrained by links to neighboring maps or ascending neuronal pathways such as the optic radiation.

How do timing selectivity and maps emerge? Many models describe how monotonic or tuned responses could arise [6, 38, 39]. Early human visual cortex exhibits monotonic responses to visual event duration and frequency [1, 2], from which tuned responses can be straightforwardly derived [40]. Our results show correlated and interacting selectivity for event duration and period (a measure of frequency). This suggests that event duration- and frequency-selective neural responses may be derived by a single mechanism [41]. Although filled and empty intervals do produce different sustained responses in the early visual cortex [42], any mechanism that captures the time between transient neural responses (which occur at both stimulus onset and offset) could give identical responses to duration and period in our stimuli. Given tuned responses, topographic maps minimize distances between similarly responding neurons, thereby increasing neural wiring efficiency [19, 20].

Timing selectivity and map organization resemble responses to visual space and to other quantities. Both visual timing and visual space show hierarchical response transformations. Selective responses and maps may facilitate these by allowing efficient interactions between similarly responding neurons. Similar encoding of timing, numerosity, and space may underlie perceptual commonalities and interactions. Similar responses, organization, and processing may underlie other cognitive functions, allowing their characterization by ultra-high-field fMRI [43].

## STAR★Methods

### Key Resources Table

**Table:** 

REAGENT or RESOURCE	SOURCE	IDENTIFIER
**Software and Algorithms**		

Vistasoft repository	https://github.com/vistalab/vistasoft	https://github.com/vistalab/vistasoft

### Lead Contact and Materials Availability

Further information and requests for resources, data and code should be directed to and will be fulfilled by the Lead Contact, Ben Harvey (b.m.harvey@uu.nl).

This study did not generate new unique reagents.

#### Experimental model and subject details

Eight right-handed subjects (one female, ages 25-35 years) participated in the experiment. Six subjects were naive to the aims of the experiment. All subjects had normal or corrected to normal vision. All subjects were briefly trained in duration discrimination tasks before scanning, to encourage attention to stimulus timing and avoid learning or habituation effects at the start of data collection. All subjects were very well educated academics, employed as PhD candidates, postdocs or professors at our university or hospital. All subjects gave written informed consent. All experimental procedures were cleared by the ethics committee of University Medical Center Utrecht.

### Method Details

#### Stimuli

We presented all visual stimuli by back-projection onto a 27.0 × 9.5 cm screen inside the MRI bore, viewed from 41cm distance. We used a digital light processing projector with no frame interpolation (Benq W6000) for its excellent temporal contrast (response time). The visible display resolution was 1600 × 538 pixels.

We displayed a large red cross over the entire image to facilitate accurate fixation at its center. Presented events consisted of a single black circle (0.4° in diameter) appearing and disappearing. These circles were placed randomly, but constrained such that the whole circle fell entirely within 0.75° of fixation and at least 0.25° from the previous circle. The display updated at 20 frame per second.

In all stimulus configurations (see main text), we presented a single event duration and period repeatedly for around 2100 ms (1 fMRI volume acquisition repetition time (TR)) before progressing to the next. We acquired 56 fMRI volumes during each stimulus configuration, over 117.6 s. We included each stimulus configuration once in a pseudo-random order within each scanning run (differing and balanced across runs), allowing their responses to be captured by a single neural response model. We tested each of 24 possible orders of all four stimulus configurations once per subject, so each subject’s data included 24 scanning runs, each totaling 470.4 s and acquired in four sessions. The subject’s task was to press a button and when a white circle was presented instead of the usual black circle. This happened pseudo-randomly, every 21 s on average, equally frequently for all timings.

The number of events presented within the 2100 ms between timing changes varied in the constant luminance, constant duration and gaps configurations. Here, event periods were not always exact factors of 2100 ms, so increments in event period sometimes fell slightly before or after 2100 ms. The maximum drift of this timing was only 300 ms, and the increments in event period were only 50 ms, so this deviation was not perceptible. The presented event timing were used for analysis.

In the constant luminance, constant duration and constant period configurations, each stimulus configuration consisted of events with durations and/or periods gradually increasing from 50-1000 ms in 50 ms steps, followed by 16.8 s with 2000 ms duration events and/or 2100 ms periods, followed by durations and/or periods gradually decreasing from 50-1000 ms in 50 ms steps, followed by 16.8 s with 2000 ms duration events and/or 2100 ms periods. In the gaps configuration, each timing progression was shorter, sampling 10 timing states rather than 20, again changing in 50 ms steps. So here we first presented events increasing duration from 50 ms to 500 ms while decreasing period from 950 ms to 500 ms, followed by 6.3 s with 50 ms duration and 2100 ms period, followed by events increasing duration from 50 ms to 500 ms while increasing period from 550 ms to 1000 ms, followed by 6.3 s with 50 ms duration and 2100 ms period, followed by events decreasing duration from 500 ms to 50 ms while increasing period from 500 ms to 950 ms, followed by 6.3 s with 50 ms duration and 2100 ms period, followed by events decreasing duration from 500 ms to 50 ms while decreasing period from 1000 ms to 550 ms, followed by 14.7 s with 50 ms duration and 2100 ms period.

The long presentation of 2000 ms duration events and/or 2100 ms periods helped to distinguish between very small response function extents (which would respond briefly and with low amplitude in the 50-1000 ms range) and very large response function extents (which respond continuously and with high amplitude) [12, 15]. This duration and period should produce little response from neural populations preferring sub-second timing. Conversely, populations whose response monotonically increases with duration, period, or mean luminance should respond most strongly here.

Repetitive stimuli like ours, rather than single events, are widely used in fMRI paradigms to maximize response amplitudes. However, repeating events affect neural responses to these events in fMRI paradigms like this, for example due to neural response adaptation. To reduce effects of adaptation on estimated timing preferences, we used a single model to capture responses to both increasing and decreasing duration and/or period progressions. This counterbalanced adaptation effects with stimuli that give both higher and lower responses preceding presentation of any timing.

#### MRI acquisition and preprocessing

We acquired MRI data on a 7T Philips Achieva scanner. Acquisition and pre-processing protocols are described fully in our previous studies [11, 13]. Briefly, we acquired T1-weighted anatomical scans, automatically segmented these with Freesurfer, then manually edited labels to minimize segmentation errors. We acquired T2^∗^-weighted functional images using a 32-channel head coil at a resolution of 1.77x1.77x1.75 mm, with 41 interleaved slices of 128x128 voxels. The resulting field of view was 227x227x72 mm. Repetition time (TR) was 2100 ms, echo time (TE) was 25 ms, flip angle was 70 degrees, and each run contained 224 TRs. We used a single shot gradient echo sequence with SENSE acceleration factor 3.0 and anterior-posterior encoding. We used a 3rd-order image-based B0 shim of the functional scan’s field of view (in-house IDL software, v6.3, RSI, Boulder, CO, USA). Scan coverage omitted anterior frontal and temporal lobes, where 7T fMRI has low response amplitudes and large spatial distortions. We typically acquired six runs in one session, depending on subject comfort, and scanned all 24 runs over 4 sessions.

We corrected for head motion artifacts between and within functional scans. We then aligned functional data from each session’s runs to anatomical scans and interpolated it into each subject’s anatomical space, allowing data from different scanning sessions to be joined together. We identified the parts of each scanning run where each stimulus configuration was presented, and averaged these together across all runs and sessions. We also separately averaged data from odd and even runs to allow cross-validation in subsequent modeling.

#### Candidate neural response models

Following previously-described approaches to investigate responses to numerosity [11, 44, 45], we fit various candidate neural response models and compared their ability to explain the observed responses to all stimulus configurations together.

There are three mathematically equivalent ways to describe the timing of the repeating events in our stimuli using two parameters (Figures S1A–S1C). First, one parameter is event duration (time from event onset to offset) and the other is event period (time from event onset to the next event onset) (Figure S1A). Second, one parameter is event duration and the other is inter-event interval (time from event offset to the next event onset) (Figure S1B). Third, one parameter is event period and the other is proportion of that period the event filled (occupancy, i.e., duty cycle) (Figure S1C). Occupancy is proportional to the mean luminance and spatial contrast in the display. Comparing model fits in these different stimulus description spaces allowed us to distinguish between predictions of parametric neural response functions in each space: we cannot distinguish between non-parametric neural response functions as these stimulus descriptions are mathematically equivalent transformations of each other.

We tested various parametric functions that might predict the relationship between the presented event’s timing and each recording site’s aggregate neural response amplitude to that event. We predict response amplitudes on a per-event basis, but these responses accumulate over a few seconds due to fMRI’s measurement of slow changes in blood flow and oxygenation. In the simplest case, the response amplitude to each event is constant with no contribution of duration. FMRI response amplitude will then increase linearly with event frequency (i.e., 1/period).

(Equation 1) Amplitude∝Constant

Next, response amplitude could increase linearly with event frequency and also independently with event duration (Figure S1D), with these two components effectively capturing transient and sustained neural response components respectively [1]. Accumulated over repeated events, the response to duration becomes equivalent to period occupancy (i.e., mean display luminance or spatial contrast).

(Equation 2) Amplitude∝Duration×AmplitudeRatio+Constant

Here, *AmplitudeRatio* is the ratio of relative amplitudes of the sustained and transient components, and is the only free parameter.

Next, the increase in response amplitude with frequency (Figure S1E) or duration (Figure S1F) may be sub-additive, so a compressive exponent is fit to capture the relationship between frequency and/or duration and response amplitude [2].

(Equation 3) $$Amplitude\propto Duration^{expDur}\times AmplitudeRatio+\frac{Frequency^{expFreq}}{Frequency}$$

Here, *expDur* and *expFreq* are exponents fit to event duration and frequency independently for each recording site. None of the response functions described so far imply any tuning for frequency and/or duration: All exhibit monotonic increases in response amplitude with increasing frequency and/or duration. Equation 3 is the general form of such monotonic response functions: amplitude increases linearly with frequency and/or duration if the exponents on frequency and/or duration are 1.

Further response functions could capture tuned responses to duration and/or period, occupancy and/or period, or duration and/or inter-event interval. Duration and period are used as examples below, but we also tested models with other stimulus description parameters (Figures S1B and S1C). The function could have a tuned Gaussian response to duration only (Figure S1G), monotonically increasing in amplitude with frequency.

(Equation 4) $$Amplitude\propto e^{-0.5\times {\left(\frac{Duration-Duration_{pref}}{\sigma }\right)}^{2}}\times \frac{Frequency^{expFreq}}{Frequency}$$

Here, *e* is the base of the natural logarithm, *Duration*_*pref*_ is the Gaussian function’s mean (i.e., the preferred duration, yielding the largest response) and σ is its standard deviation.

Finally, the function could have a 2-dimensional Gaussian tuning to both duration and period, together with a compressive increase in response amplitude with frequency.

(Equation 5) $$X=\left(Duration-Duration_{pref}\right)\times \cos\left(\theta \right)-\left(Period-Period_{pref}\right)\times \sin\left(\theta \right)$$

(Equation 6) $$Y=\left(Duration-Duration_{pref}\right)\times \sin\left(\theta \right)+\left(Period-Period_{pref}\right)\times \cos\left(\theta \right)$$

(Equation 7) $$Amplitude\propto e^{-0.5\times \left({\left(\frac{Y}{\sigma_{maj}}\right)}^{2}+{\left(\frac{X}{\sigma_{min}}\right)}^{2}\right)}\times \frac{Frequency^{expFreq}}{Frequency}$$

Here, θ is the angulation of the Gaussian function’s major axis, *σ*_*maj*_ and *σ*_*min*_ are its standard deviation along its major and minor axes respectively. If these standard deviations are equal, the Gaussian is circular symmetric (Figure S1H). Otherwise, it is anisotropic and angulated (Figures 1B, 2A, and S1I).

Finally, we tested whether responses to both timing parameters could be captured by a model tuned to temporal frequency, as early visual areas contain temporal frequency-tuned neurons. We determining the Fourier frequency power spectrum of the display’s luminance time course and fit a Gaussian function to this. We repeated this for a single response to the power spectrum within each TR, and also for a response to the power spectrum with every event offset.

#### Neural response model fitting and comparison

The resulting candidate neural response functions (Figures S1D–S1I) each predicted how each recording site’s response amplitude changed with stimulus event timing. We used forward modeling to convert these response amplitude predictions to fMRI response time course predictions, and compared these to measured responses following population receptive field (pRF) modeling approaches [15]. For each recording site on the cortical surface, we repeated this for the average of all data and the two cross-validation splits. We tested predictions from a large set of candidate combinations of the free parameters for each candidate neural response model, followed by gradient descent to search between the tested combinations.

At the time of the offset of every stimulus event (Figure 1A), we evaluated the candidate neural response function (Figure 1B) at the presented timing of that event (Figure 1C). We repeated this for the whole stimulus time course in all stimulus configurations to give a predicted neural response amplitude time course for the whole stimulus sequence (Figure 1D). Where a compressive exponent on event frequency was included, we scaled this prediction following the instantaneous event frequency (Figure 1E). We then convolved this prediction with a hemodynamic response function (HRF) to give a predicted fMRI response time course (Figure 1F) for this candidate neural response function. We determined the correlation between this prediction and the measured fMRI response time course (Figure 1G) at each recording site. We then fit the free parameters of the model to maximize the correlation between this prediction and the measured fMRI response time course.

We used cross-validation to compare the prediction of different candidate response models despite the different numbers of free parameters. We took the best-fitting parameter set from the odd numbered scans and quantified the correlation between its prediction and the measured fMRI time course from the even numbered scans, and vice versa, giving the cross-validated variance explained by each candidate model.

For the best fitting model, we then estimated subject-specific HRF parameters [46] across the whole acquired fMRI volume from all the data recorded from each subject, as described elsewhere [33]. This avoids systematic over- or underestimation of response function extents resulting from inaccurate HRF parameters (faster or slower than the subject’s, respectively). However, this procedure does not systematically affect estimated timing preferences because these are fit to responses to stimulus progressions in two opposite directions. Having fit each subject’s HRF parameters, we then re-fit the response model using these HRF parameters. We used the resulting neural response model’s parameters for all further analyses.

Candidate preferred durations and periods extended beyond the presented timing range. Therefore, fit parameters within the stimulus range were not just the best fit of a limited set. However, we could not accurately estimate fit parameters outside of the stimulus range, so excluded any recording sites with preferred durations or periods outside this range from further analysis.

#### Region of interest definitions

We rendered the variance explained by the neural response model onto the cortical surface. This highlighted ten localized increases in variance explained with consistent locations relative to major sulci, which formed the basis of our regions of interest (ROIs). We define our map ROIs by taking the variance explain values for each vertex on the cortical surface model and performing surface-based clustering on these values. We repeated this at several thresholds of variance explained, finding a range of variance explained values in each subject that produced the same cluster count. We then clustered variance explained values thresholded at the bottom of this range (the knee), with these clusters forming our ROIs. In some cases, we merged two adjacent clusters into a single ROI, or split a single large cluster into two parts where it contained two contiguous maps (common in TTOP/TTOA and TPCS/TPCM).

We then rendered each recording site’s preferred duration (Figures 3B, 3C, S2, and S3) onto the cortical surface. In each map ROI, we visually defined lines joining locations at map edges with similar preferred duration at the low and high ends of the preferred timing range seen in the map (the ‘ends’ of the map). These end lines allowed us to quantify how preferred duration and period changed with distance across each map (Figures 4 and S4). However, because the direction of timing progressions were somewhat variable between subjects [11], this approach relies on locations of different timing preferences to analyze changes in timing preferences with cortical location, and is therefore circular. We also looked for changes in the response functions’ major and the minor extents with preferred duration within each map (Figure 5).

We converted each individual subject’s ROI locations to Montreal Neurological Institute (MNI) x, y, and z coordinates to locate them in an average brain. We transformed each subject’s anatomical MRI data, together with the locations of each ROI’s center on the cortical surface, into MNI average template space [47] with MINC’s ‘mincresample’ tool (http://packages.bic.mni.mcgill.ca) using rigid alignment and linear scaling. We took the mean and standard deviation of the resulting MNI coordinates of each map across subjects (Table S1). This volumetric approach does not allow us to locate maps on the cortical surface to show their overlap. Therefore we also transformed each subject’s anatomical MRI data, together with the map surfaces and centers to the Talairach N27 surface using AFNI’s 3dAllineate and 3dNwarpApply tools. This gives the template brains shown in Figure 3A and the Talairach coordinates in Table S1.

#### Analysis of changes within each ROI

To calculate distances along each timing map, we determined the distance along the cortical surface from each point in each ROI to the nearest point on the lines of the lowest and highest preferred duration. The ratio between the distances to each end line gave a normalized distance along the ROI in the primary direction of change of timing preferences. We multiplied this by the mean ROI length in this direction to give a cortical distance measure in millimeters. We binned the recording points within every 2 mm along each timing map, calculating the mean and standard error of their preferred durations and periods in the full data and the two cross-validation splits (Figures 4 and S4). In calculating standard errors, we corrected for up-sampling of data onto our cortical surface model. Bins were excluded if their cortical surface extent of the recording sites they contained was smaller than one fMRI voxel (1.77 mm^2^) or smaller than the point spread function of cortical 7T fMRI (2 mm^2^) [48]. We fit logarithmic functions to bootstrapped samples of the bin means. From these bootstrapped fits, we took the median slope and intercept as the best fitting progressions of preferred duration and period. We determined 95% confidence intervals by plotting all bootstrapped fit lines and finding the 2.5% and 97.5% percentiles of their values.

For each ROI we also looked for changes in the response functions’ major and the minor extents with preferred duration, in data from the same map grouped across subjects (Figure 5). To visualize these changes, we binned the recording sites within every 50 ms increase in preferred duration, calculating the mean and standard error of the response function extent. Bins were again excluded if their cortical surface extent was smaller than one fMRI voxel or the point spread function of cortical 7T fMRI.

#### Analysis of changes between ROIs

We also compared several properties of these maps and their responses between maps. For each map in each hemisphere of each subject, we quantified the map’s cortical surface area (Figure 6A). We then quantified the mean of several properties across the recording sites within the map: model variance explained, compressive exponent (Figure 6B), preferred duration (Figure 6C) and period, the orientation of the response function’s major axis (Figure 6F), the extent of the response function along its major and minor axes (Figure 6G), and the aspect ratio of the response function (i.e., major axis extent / minor axis extent) (Figure 6H). Finally, we quantified the interquartile range of preferred durations (Figure 6D) and preferred periods (Figure 6E) of recording sites within each map.

#### Numerosity mapping

We acquired numerosity mapping responses to examine the relationship between timing map and numerosity map positions. The numerosity mapping paradigm was identical to that described in previous studies [11, 13], although with a smaller set of stimulus configurations (‘constant area’ and ‘constant dot size’) in subjects 1, 4, 5, 6, 7 and 8. The stimulus consisted of dot patterns that gradually changed their numerosity over time. The stimulus had a radius of 0.75°, the same as the timing stimuli. Again, subjects responded when dot patterns were white instead of black (> 80% accuracy), and a large diagonal cross was presented to aid accurate fixation.

Functional runs were each 182 time frames (382.2 s) in duration, of which the first 6 time frames (12.6 s) were discarded to ensure a steady state of data acquisition and neural responses to stimuli. Data were analyzed following procedures described in detail elsewhere [11, 12], which closely follow the analyses described above for characterizing responses to event timing.

#### Visual field mapping

We acquired visual field mapping responses to examine the relationship between timing map and visual field map positions. The visual field mapping paradigm was identical to that described in previous studies [11, 13]. The stimulus consisted of drifting bar apertures at various orientations, which exposed a moving checkerboard pattern. The stimulus had a radius of 6.35°, larger than the timing mapping stimuli (0.75° radius). Two diagonal red lines, intersecting at the center of the display, were again presented throughout the entire scanning run. Subjects pressed a button when these lines changed color, and responded on 80%–100% of color changes within each scanning run.

Functional runs were each 182 time frames (382.2 s) in duration, of which the first 6 time frames (12.6 s) were discarded to ensure a steady state of data acquisition and neural responses to stimuli. Visual field mapping data were analyzed following a standard population receptive field analysis, as described elsewhere [15, 33]. This gave the preferred visual field position of each recording site, from which we calculated the eccentricity and polar angle. We identified visual field map borders based on reversals in polar angle and eccentricity of visual field position preference and identified particular visual field maps with reference to previous studies [17, 36, 49, 50].

### Quantification and Statistical Analysis

All statistical analyses were performed in MATLAB, with significance defined at p < 0.05. Results of specific tests are stated with summary statistics and noting the test used in the Results.

#### Neural response model comparison

We compared the cross-validated variance explained between candidate response models in all recording sites with at least 20% variance explained by one candidate model. For each subject, we took each map’s mean variance explained from all models, and compared these using paired t tests (Figures S1J and S1K). To demonstrate generalization of these effects across subjects, we similarly took each subject’s mean variance explained from all models, and compared these using paired t tests.

We converted the variance explained measures from our neural response models to probabilities (of observing these model fits by chance) by fitting the same response model to recordings from 163,131 white matter recording sites in the same scans. We then determined the proportion this null distribution exceeding any variance explained [13]. We excluded recording sites where models explained below 10% of response variance (a probability above 0.03) from further analysis.

#### Analysis of changes within each ROI

To quantify the statistical significance of duration and period progressions across the cortical surface within each map, we correlated preferred duration and preferred period against the recording site’s cortical distance along the map (Figure S4). When converting correlation coefficients to p values, we corrected for up-sampling of data onto our cortical surface model by dividing the number of cortical surface vertices by the up-sampling factor. We used false discovery rate (FDR) correction for multiple comparisons [51], taking probabilities from all maps in all subjects into account. Because of the circular nature of this analysis, we repeated this analysis for models fit to independent splits of odd and even scan runs (to test the repeatability of these progressions) and tested the correlation between timing preferences on these two halves of the data (which is not circular, but ignores the cortical locations of recording sites).

To quantify the relationship between preferred durations and periods, we correlated these preferences across the recording sites within each map, again correcting for upsampling and FDR.

Plotting the relationship between the response functions’ extent and preferred duration revealed that response function extent first increased with preferred duration, and second increased as the preferred duration moved away from the middle of the presented range (Figure 5). To test both progressions independently, it was necessary to include the same number of recording sites with preferred durations on either side of the middle of the presented range. Therefore, we first split the recording sites into those with preferred durations above and below the middle of the presented range. From the larger group, we repeatedly discarded a random selection so both groups had the same count, following a bootstrap procedure. We then fit a general linear model to this combined data, with three predictors: preferred duration; the absolute difference between the preferred duration and the middle of the presented range; and a constant to capture the intercept of the progressions. We repeated this procedure in 1000 bootstrap permutations, each permutation discarding a different random selection. We took the mean t-statistic for each predictor across permutations, and converted this to a probability taking into account the (fixed) number of recording sites, corrected for upsampling as already described. We repeated this procedure for the bin means in Figure 5 to give the best fitting lines. We determined 95% confidence intervals by plotting fit lines from all permutations and finding the 2.5% and 97.5% percentiles of their values.

#### Analysis of changes between ROIs

We also compared several properties of the timing maps and their responses between maps. For each map in each hemisphere of each subject, we quantified the map’s cortical surface area (Figure 6A). We then quantified the mean of several properties across the recording sites within the map: model variance explained, compressive exponent (Figure 6B), preferred duration (Figure 6C) and period, the orientation of the response function’s major axis (Figure 6F), the extent of the response function along its major and minor axes (Figure 6G), and the aspect ratio of the response function (i.e., major axis extent / minor axis extent) (Figure 6H). Finally, we quantified the interquartile range of preferred durations (Figure 6D) and preferred periods (Figure 6E) of recording sites within each map.

We compared each of these measures between maps using separate three-way analyses of variance (ANOVAs), with hemisphere, map and subject as factors (n = 155). For measures where there was no effect of hemisphere in this three-way ANOVA (mean and interquartile range of preferred durations and period, mean major and minor axis extents, mean aspect ratio and mean response function orientation), we used a two-way ANOVA with map and subject as factors. Where these ANOVAs revealed significant differences between maps, we tested where these differences reach significance using subsequent multiple comparison tests [52, 53].

### Data and Code Availability

The code generated during this study is available in the Vistasoft repository (https://github.com/vistalab/vistasoft).

The datasets supporting the current study have not yet been deposited in a public repository because of data protection issues, but are available from the corresponding author on request.

## Supplemental Information

Document S1. Figures S1–S7 and Table S1

Document S2. Article plus Supplemental Information

## References

[1] Stigliani A., Jeska B., Grill-Spector K. (2017). Encoding model of temporal processing in human visual cortex. Proc. Natl. Acad. Sci. USA.

[2] Zhou J., Benson N.C., Kay K.N., Winawer J. (2018). Compressive temporal summation in human visual cortex. J. Neurosci..

[3] Merchant H., Pérez O., Zarco W., Gámez J. (2013). Interval tuning in the primate medial premotor cortex as a general timing mechanism. J. Neurosci..

[4] Merchant H., Zarco W., Pérez O., Prado L., Bartolo R. (2011). Measuring time with different neural chronometers during a synchronization-continuation task. Proc. Natl. Acad. Sci. USA.

[5] Heron J., Aaen-Stockdale C., Hotchkiss J., Roach N.W., McGraw P.V., Whitaker D. (2012). Duration channels mediate human time perception. Proc. Biol. Sci..

[6] Buonomano D.V., Merzenich M.M. (1995). Temporal information transformed into a spatial code by a neural network with realistic properties. Science.

[7] Bruno A., Cicchini G.M. (2016). Multiple channels of visual time perception. Curr. Opin. Behav. Sci..

[8] Walsh V. (2003). A theory of magnitude: common cortical metrics of time, space and quantity. Trends Cogn. Sci..

[9] Nieder A., Freedman D.J., Miller E.K. (2002). Representation of the quantity of visual items in the primate prefrontal cortex. Science.

[10] Nieder A., Miller E.K. (2004). A parieto-frontal network for visual numerical information in the monkey. Proc. Natl. Acad. Sci. USA.

[11] Harvey B.M., Dumoulin S.O. (2017). A network of topographic numerosity maps in human association cortex. Nat. Hum. Behav..

[12] Harvey B.M., Klein B.P., Petridou N., Dumoulin S.O. (2013). Topographic representation of numerosity in the human parietal cortex. Science.

[13] Harvey B.M., Fracasso A., Petridou N., Dumoulin S.O. (2015). Topographic representations of object size and relationships with numerosity reveal generalized quantity processing in human parietal cortex. Proc. Natl. Acad. Sci. USA.

[14] Protopapa F., Hayashi M.J., Kulashekhar S., van der Zwaag W., Battistella G., Murray M.M., Kanai R., Bueti D. (2019). Chronotopic maps in human supplementary motor area. PLoS Biol..

[15] Dumoulin S.O., Wandell B.A. (2008). Population receptive field estimates in human visual cortex. Neuroimage.

[16] Wandell B.A., Brewer A.A., Dougherty R.F. (2005). Visual field map clusters in human cortex. Philos. Trans. R. Soc. Lond. B Biol. Sci..

[17] Amano K., Wandell B.A., Dumoulin S.O. (2009). Visual field maps, population receptive field sizes, and visual field coverage in the human MT+ complex. J. Neurophysiol..

[18] Dumoulin S.O., Bittar R.G., Kabani N.J., Baker C.L., Le Goualher G., Bruce Pike G., Evans A.C. (2000). A new anatomical landmark for reliable identification of human area V5/MT: a quantitative analysis of sulcal patterning. Cereb. Cortex.

[19] Chen B.L., Hall D.H., Chklovskii D.B. (2006). Wiring optimization can relate neuronal structure and function. Proc. Natl. Acad. Sci. USA.

[20] Durbin R., Mitchison G. (1990). A dimension reduction framework for understanding cortical maps. Nature.

[21] Kay K.N., Winawer J., Mezer A., Wandell B.A. (2013). Compressive spatial summation in human visual cortex. J. Neurophysiol..

[22] Goldberg G. (1985). Supplementary motor area structure and function: review and hypotheses. Behav. Brain Sci..

[23] Romo R., Schultz W. (1987). Neuronal activity preceding self-initiated or externally timed arm movements in area 6 of monkey cortex. Exp. Brain Res..

[24] Hayashi M.J., Kanai R., Tanabe H.C., Yoshida Y., Carlson S., Walsh V., Sadato N. (2013). Interaction of numerosity and time in prefrontal and parietal cortex. J. Neurosci..

[25] Hayashi M.J., Ditye T., Harada T., Hashiguchi M., Sadato N., Carlson S., Walsh V., Kanai R. (2015). Time adaptation shows duration selectivity in the human parietal cortex. PLoS Biol..

[26] Hayashi M.J., van der Zwaag W., Bueti D., Kanai R. (2018). Representations of time in human frontoparietal cortex. Commun. Biol..

[27] Bueti D., Bahrami B., Walsh V. (2008). Sensory and association cortex in time perception. J. Cogn. Neurosci..

[28] Wang J., Narain D., Hosseini E.A., Jazayeri M. (2018). Flexible timing by temporal scaling of cortical responses. Nat. Neurosci..

[29] Jazayeri M., Shadlen M.N. (2015). A neural mechanism for sensing and reproducing a time interval. Curr. Biol..

[30] Leon M.I., Shadlen M.N. (2003). Representation of time by neurons in the posterior parietal cortex of the macaque. Neuron.

[31] Harvey B.M., Ferri S., Orban G.A. (2017). Comparing parietal quantity-processing mechanisms between humans and macaques. Trends Cogn. Sci..

[32] Harvey B.M. (2016). Quantity cognition: numbers, numerosity, zero and mathematics. Curr. Biol..

[33] Harvey B.M., Dumoulin S.O. (2011). The relationship between cortical magnification factor and population receptive field size in human visual cortex: constancies in cortical architecture. J. Neurosci..

[34] Gibbon J., Malapani C., Dale C.L., Gallistel C. (1997). Toward a neurobiology of temporal cognition: advances and challenges. Curr. Opin. Neurobiol..

[35] Jazayeri M., Shadlen M.N. (2010). Temporal context calibrates interval timing. Nat. Neurosci..

[36] Hagler D.J., Sereno M.I. (2006). Spatial maps in frontal and prefrontal cortex. Neuroimage.

[37] Mackey W.E., Winawer J., Curtis C.E. (2017). Visual field map clusters in human frontoparietal cortex. eLife.

[38] Ivry R.B., Schlerf J.E. (2008). Dedicated and intrinsic models of time perception. Trends Cogn. Sci..

[39] Karmarkar U.R., Buonomano D.V. (2007). Timing in the absence of clocks: encoding time in neural network states. Neuron.

[40] Dehaene S., Changeux J.P. (1993). Development of elementary numerical abilities: a neuronal model. J. Cogn. Neurosci..

[41] Hartcher-O’Brien J., Brighouse C., Levitan C.A. (2016). A single mechanism account of duration and rate processing via the pacemaker-accumulator and beat frequency models. Curr. Opin. Behav. Sci..

[42] Iwasaki M., Noguchi Y., Kakigi R. (2019). Neural correlates of time distortion in a preaction period. Hum. Brain Mapp..

[43] Dumoulin S.O., Fracasso A., van der Zwaag W., Siero J.C.W., Petridou N. (2018). Ultra-high field MRI: advancing systems neuroscience towards mesoscopic human brain function. Neuroimage.

[44] Harvey B.M., Dumoulin S.O. (2017). Can responses to basic non-numerical visual features explain neural numerosity responses?. Neuroimage.

[45] Harvey B.M., Dumoulin S.O. (2017). Data describing the accuracy of non-numerical visual features in predicting fMRI responses to numerosity. Data Brief.

[46] Handwerker D.A., Ollinger J.M., D’Esposito M. (2004). Variation of BOLD hemodynamic responses across subjects and brain regions and their effects on statistical analyses. Neuroimage.

[47] Collins D.L., Neelin P., Peters T.M., Evans A.C. (1994). Automatic 3D intersubject registration of MR volumetric data in standardized Talairach space. J. Comput. Assist. Tomogr..

[48] Shmuel A., Yacoub E., Chaimow D., Logothetis N.K., Ugurbil K. (2007). Spatio-temporal point-spread function of fMRI signal in human gray matter at 7 Tesla. Neuroimage.

[49] Larsson J., Heeger D.J. (2006). Two retinotopic visual areas in human lateral occipital cortex. J. Neurosci..

[50] Swisher J.D., Halko M.A., Merabet L.B., McMains S.A., Somers D.C. (2007). Visual topography of human intraparietal sulcus. J. Neurosci..

[51] Chumbley J., Worsley K., Flandin G., Friston K. (2010). Topological FDR for neuroimaging. Neuroimage.

[52] Hochberg Y., Tamhane A.C. (1987). Multiple Comparison Procedures.

[53] Searle S.R., Speed F.M., Milliken G.A. (1980). Population marginal means in the linear model: an alternative to least squares means. Am. Stat..

