# Supplementary material for: A Network of Topographic Maps in Human Association Cortex Hierarchically Transforms Visual Timing-Selective Responses
Source: Curr Biol. 2020 Apr 20;30(8):1424–1434.e6. doi: 10.1016/j.cub.2020.01.090 (PMC7181178; doi:10.1016/j.cub.2020.01.090)
Supplement: Document S1. Figures S1–S7 and Table S1 [file mmc1.pdf]

Current Biology, Volume *30*

Supplemental Information

A Network of Topographic Maps in Human Association Cortex Hierarchically Transforms Visual Timing-Selective Responses

Ben M. Harvey, Serge O. Dumoulin, Alessio Fracasso, and Jacob M. Paul

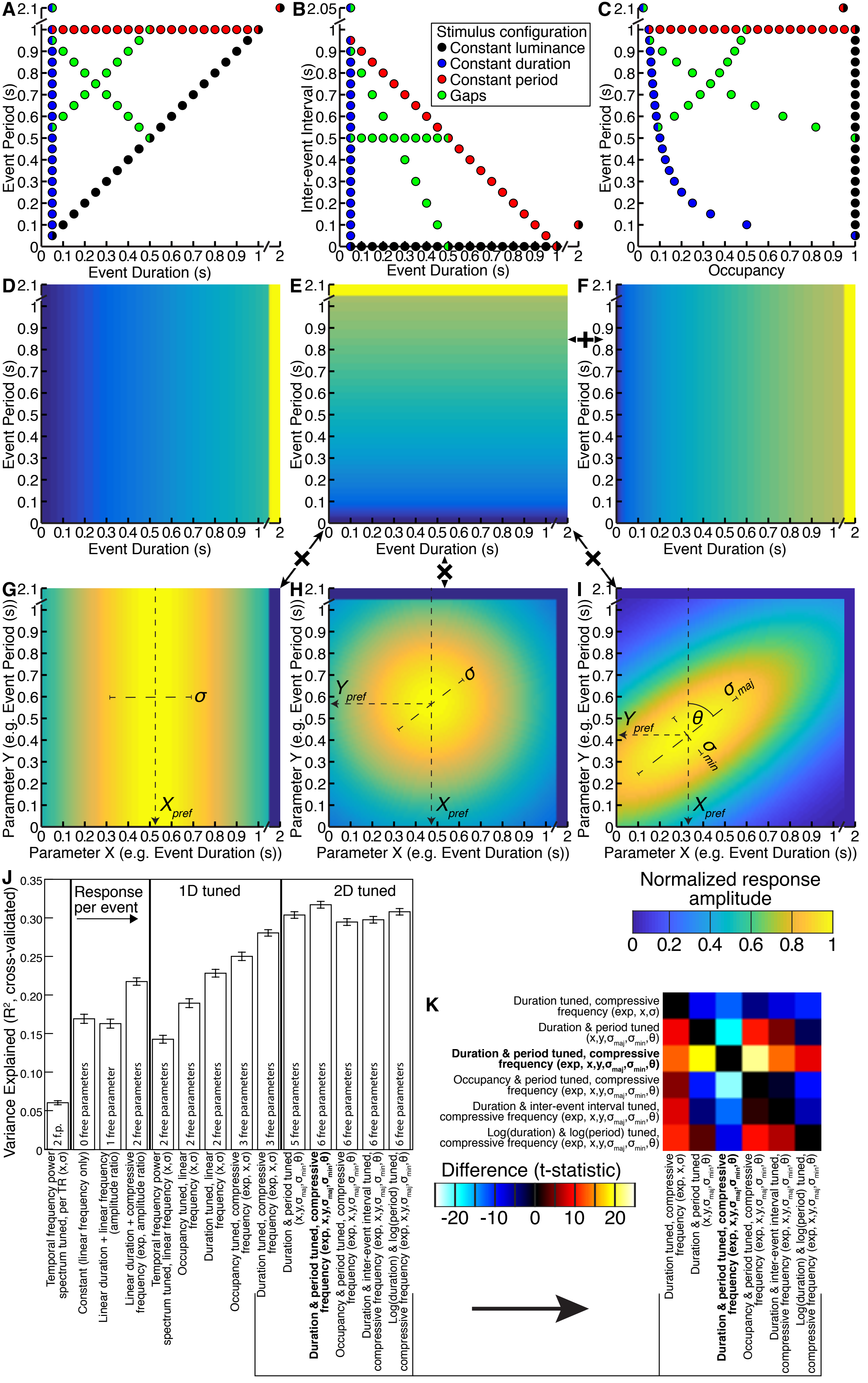

A
B
C
D
E
F
G
H
I
J
K
Stimulus configuration
Constant luminance
Constant duration
Constant period
Gaps
Event Period (s)
Inter-event Interval (s)
Event Duration (s)
Occupancy
Parameter Y (e.g. Event Period (s))
Parameter X (e.g. Event Duration (s))
σ
X_{pref}
Y_{pref}
$σ_{maj}$
$σ_{min}$
θ
Normalized response amplitude
Variance Explained (R², cross-validated)
Response per event
1D tuned
2D tuned
2 f.p.
0 free parameters
1 free parameter
2 free parameters
3 free parameters
5 free parameters
6 free parameters
Temporal frequency power spectrum tuned, per TR (x,σ)
Constant (linear frequency only)
Linear duration + linear frequency (amplitude ratio)
Linear duration + compressive frequency (exp, amplitude ratio)
Temporal frequency power spectrum tuned, linear frequency (x,σ)
Occupancy tuned, linear frequency (x,σ)
Duration tuned, linear frequency (x,σ)
Occupancy tuned, compressive frequency (exp, x,σ)
Duration tuned, compressive frequency (exp, x,σ)
Duration & period tuned (x,y,$σ_{maj}$,$σ_{min}$,θ)
Duration & period tuned, compressive frequency (exp, x,y,$σ_{maj}$,$σ_{min}$,θ)
Occupancy & period tuned, compressive frequency (exp, x,y,$σ_{maj}$,$σ_{min}$,θ)
Duration & inter-event interval tuned, compressive frequency (exp, x,y,$σ_{maj}$,$σ_{min}$,θ)
Log(duration) & log(period) tuned, compressive frequency (exp, x,y,$σ_{maj}$,$σ_{min}$,θ)
Difference (t-statistic)

Figure S1. Candidate timing representations and model fits. Related to Figure 1. (**A**) There are several mathematically equivalent ways to describe the timing of the repeating events in our stimulus. First, we can determine the event's duration and its period. Event duration cannot be greater than period as this would require multiple events to happen simultaneously. (**B**) Second, we can determine the event's duration and its inter-event interval. (**C**) Finally, we can determine the event's occupancy and its period. (**D**) In the simplest parametric models, the amplitude of the response to each event may monotonically and linearly increase with event duration (Equation 2) or be constant for all event durations (Equation 1, not shown). (**E**) Alternatively, the response amplitude to each event may be sub-additive, so more frequency events each produce weaker responses. This can be captured by fitting a variable compressive exponent parameter on event frequency (0.5 is shown here), in Equation 3. (**F**) A similar compressive exponent on event duration (0.5 is shown here) could capture sub-additive accumulation of response amplitude with event duration, potentially with a weighted sum (+) of the sub-additive response to frequency also contributing. (**G**) Alternatively, responses may display Gaussian tuning to one parameter (for example duration) and be unaffected by the other parameter (for example period). (**H**) The response may display circular symmetric Gaussian tuning for both parameters. (**I**) Finally, the response may display anisotropic Gaussian tuning for both parameters. A sub-additive accumulation of responses with event frequency (×) can be combined with the Gaussian function in (G) following Equations 4, or with the Gaussian functions in (H, I) following Equation 5. (**J**) We compared the ability of these different models to predict the responses observed. We fit the parameters of each model in one half of our scans and determined the proportion of the variance in the complementary half that the resulting model explained. This revealed that models tuned to event duration and period, with a compressive accumulation of response amplitude with event frequency (labelled in bold text) best captured the observed responses. (**K**) T-statistics of paired comparisons between the variance explained in cross-validated data by the best fitting models.

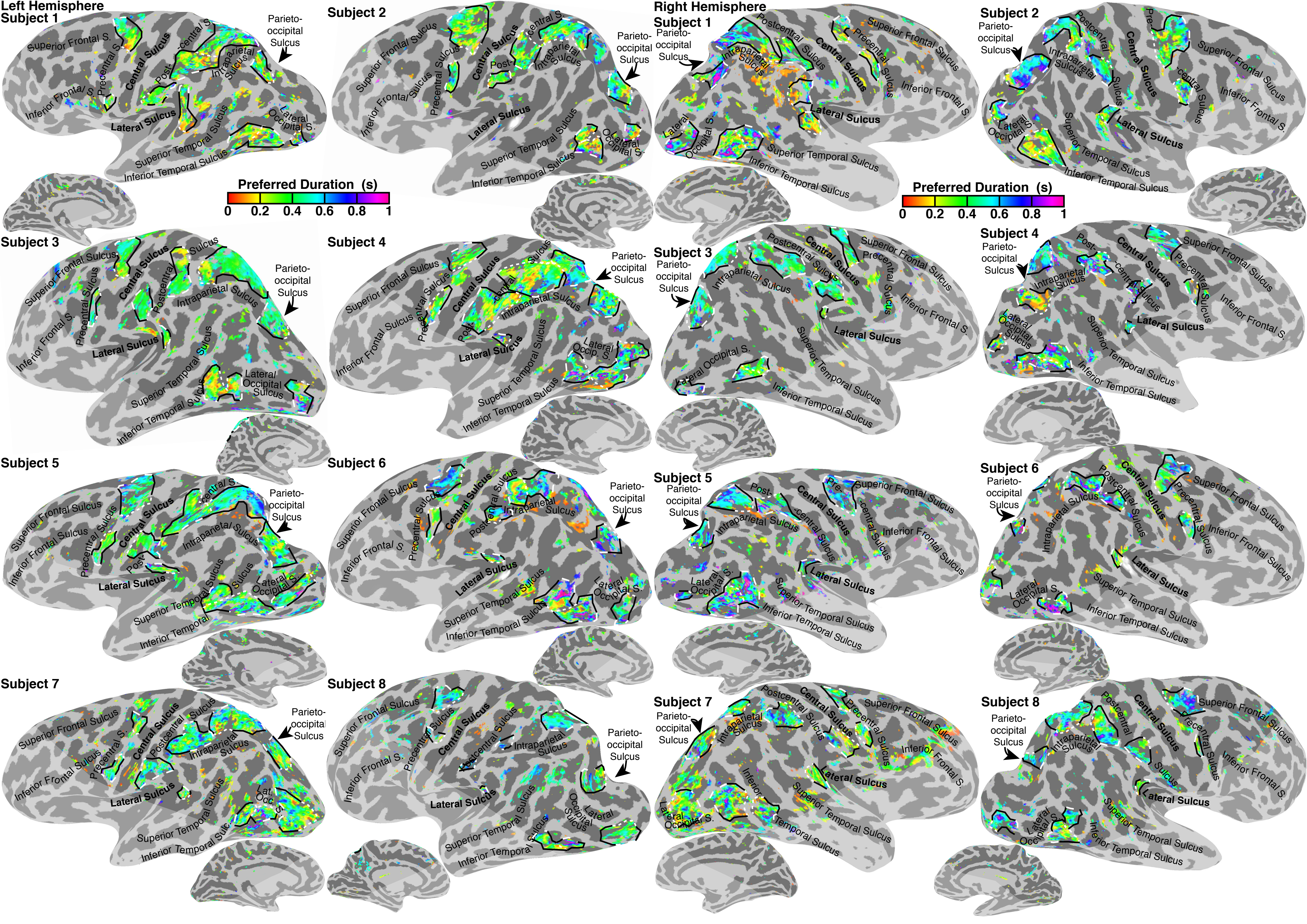
Left Hemisphere
Subject 1
Superior Frontal S.
Precentral S.
Central Sulcus
Post-
central S.
Intraparietal Sulcus
Parieto-
occipital
Sulcus
Inferior Frontal S.
Lateral Sulcus
Superior Temporal Sulcus
Inferior Temporal Sulcus
Lateral Occipital S.
Preferred Duration (s)
0
0.2
0.4
0.6
0.8
1
Subject 2
Superior Frontal Sulcus
Inferior Frontal Sulcus
Precentral Sulcus
Central Sulcus
Post
central S.
Intraparietal Sulcus
Parieto-
occipital
Sulcus
Lateral Sulcus
Superior Temporal Sulcus
Inferior Temporal Sulcus
Lateral Occipital S.
Right Hemisphere
Subject 1
Parieto-
occipital
Sulcus
Postcentral Sulcus
Intraparietal Sulcus
Central Sulcus
Precentral Sulcus
Superior Frontal Sulcus
Inferior Frontal S.
Lateral Sulcus
Lateral Occipital S.
Superior Temporal Sulcus
Inferior Temporal Sulcus
Subject 2
Parieto-
occipital
Sulcus
Postcentral Sulcus
Pre-
Intraparietal Sulcus
Central Sulcus
central Sulcus
Superior Frontal Sulcus
Inferior Frontal S.
Lateral Sulcus
Lateral Occipital S.
Superior Temporal Sulcus
Inferior Temporal Sulcus
Preferred Duration (s)
0
0.2
0.4
0.6
0.8
1
Subject 3
Superior Frontal Sulcus
Central Sulcus
Sulcus
Postcentral
Intraparietal Sulcus
Parieto-
occipital
Sulcus
Inferior Frontal S.
Precentral Sulcus
Lateral Sulcus
Superior Temporal Sulcus
Lateral Occipital Sulcus
Inferior Temporal Sulcus
Subject 4
Superior Frontal Sulcus
Inferior Frontal Sulcus
Precentral Sulcus
Central Sulcus
Post central
Intraparietal Sulcus
Sulcus
Parieto-
occipital
Sulcus
Lateral Sulcus
Lateral Occip. S.
Superior Temporal Sulcus
Inferior Temporal Sulcus
Subject 3
Parieto-
occipital
Sulcus
Intraparietal Sulcus
Postcentral Sulcus
Central Sulcus
Precentral Sulcus
Superior Frontal Sulcus
Inferior Frontal S.
Lateral Sulcus
Lateral Occipital S.
Superior Temporal Sulcus
Inferior Temporal Sulcus
Subject 4
Parieto-
occipital
Sulcus
Post-
Intraparietal Sulcus
central Sulcus
Central Sulcus
Precentral Sulcus
Superior Frontal Sulcus
Inferior Frontal S.
Lateral Sulcus
Lateral Occipital Sulcus
Superior Temporal Sulcus
Inferior Temporal Sulcus
Subject 5
Superior Frontal Sulcus
Inferior Frontal Sulcus
Precentral Sulcus
Central Sulcus
central S.
Post
Intraparietal Sulcus
Parieto-
occipital
Sulcus
Lateral Sulcus
Superior Temporal Sulcus
Sulcus
Lateral Occipital S.
Inferior Temporal
Subject 6
Superior Frontal Sulcus
Inferior Frontal S.
Precentral Sulcus
Central Sulcus
Postcentral Sulcus
Intraparietal Sulcus
Parieto-
occipital
Sulcus
Lateral Sulcus
Superior Temporal Sulcus
Inferior Temporal Sulcus
Lateral Occipital S.
Subject 5
Parieto-
occipital
Sulcus
Post-
Intraparietal Sulcus
Central Sulcus
Pre-
central Sulcus
Superior Frontal Sulcus
Inferior Frontal Sulcus
Lateral Sulcus
Lateral Occipital S.
Superior Temporal Sulcus
Inferior Temporal Sulcus
Subject 6
Parieto-
occipital
Sulcus
Intraparietal Sulcus
Postcentral Sulcus
Central Sulcus
Precentral Sulcus
Superior Frontal Sulcus
Inferior Frontal Sulcus
Lateral Sulcus
Lateral Occipital S.
Superior Temporal Sulcus
Inferior Temporal Sulcus
Subject 7
Superior Frontal Sulcus
Inferior Frontal Sulcus
Precentral S.
Central Sulcus
Postcentral Sulcus
Intraparietal Sulcus
Parieto-
occipital
Sulcus
Lateral Sulcus
Superior Temporal Sulcus
Lat. Occ. S.
Superior Temporal Sulc.
Inferior Temporal Sulc.
Subject 8
Superior Frontal Sulcus
Inferior Frontal S.
Precentral Sulcus
Central Sulcus
Postcentral Sulcus
Intraparietal Sulcus
Parieto-
occipital
Sulcus
Lateral Sulcus
Superior Temporal Sulcus
Occipital Sulcus
Lateral
Inferior Tempora
Subject 7
Parieto-
occipital
Sulcus
Intraparietal Sulcus
Postcentral Sulcus
Central Sulcus
Precentral Sulcus
Superior Frontal Sulcus
Inferior Frontal S.
Lateral Sulcus
Inferior
Temporal Sulcus
Superior Temporal Sulcus
Lateral Occipital S.
Subject 8
Parieto-
occipital
Sulcus
Intraparietal Sulcus
Postcentral
Central Sulcus
Sulcus
Precentral Sulcus
Superior Frontal Sulcus
Inferior Frontal S.
Lateral Sulcus
Lateral Occipital S.
Superior Temporal Sulcus
Inferior Temporal Sulcus

Figure S2. Preferred duration of each recording site on the cortical surface for all subjects. Related to Figure 3B. Cortical surface anatomy in grays, major sulci labelled black. Recording sites with over 10% response variance explained by the response model are shown. Timing maps outlined in black and dashed white lines, following Figure 3B.

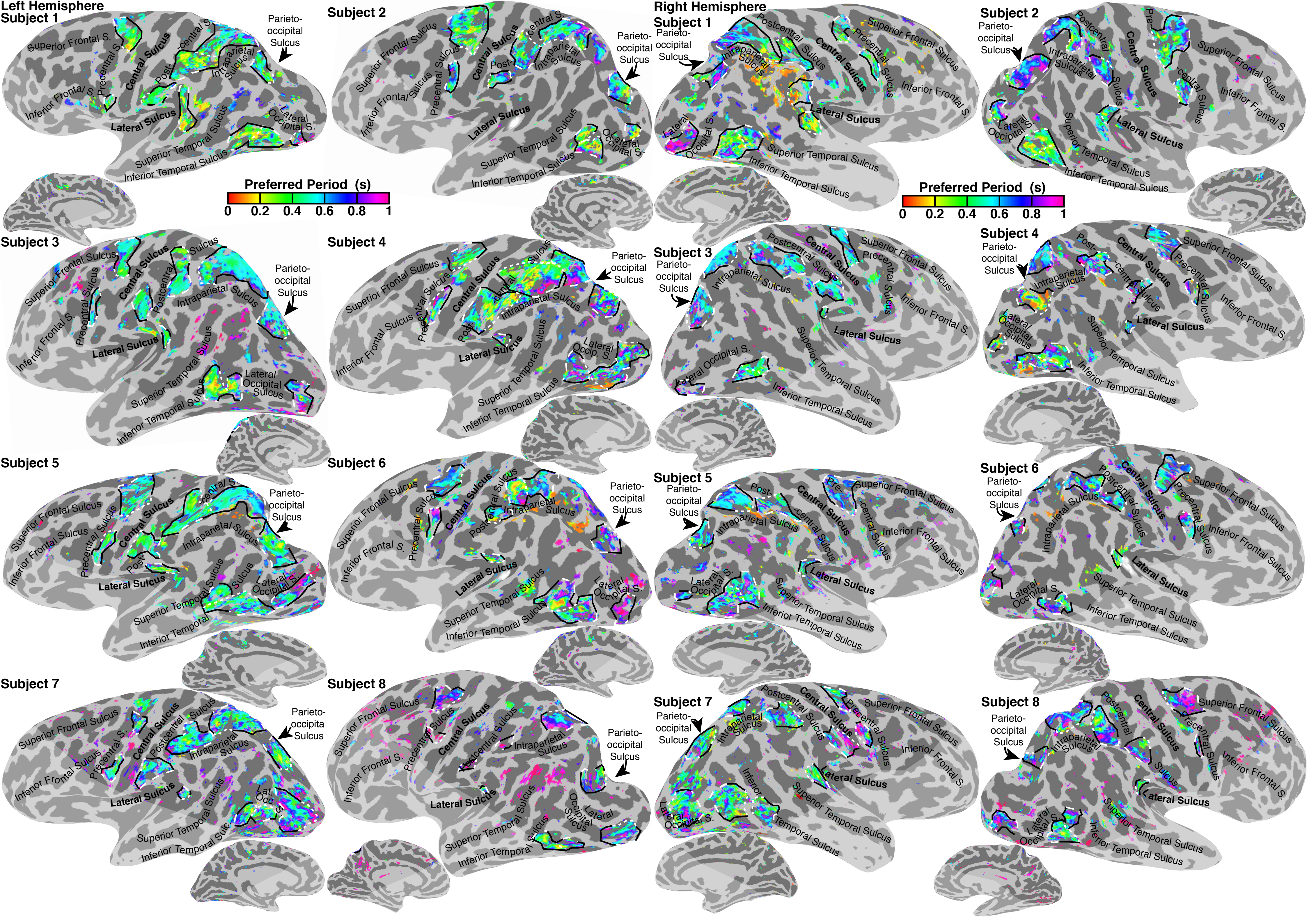

Left Hemisphere
Subject 1
Superior Frontal S.
Precentral S.
Central Sulcus
Post-central S.
Intraparietal Sulcus
Parieto-occipital Sulcus
Inferior Frontal S.
Lateral Sulcus
Superior Temporal Sulcus
Inferior Temporal Sulcus
Lateral Occipital S.
Subject 2
Preferred Period (s)
0
0.2
0.4
0.6
0.8
1
Right Hemisphere
Subject 3
Subject 4
Subject 5
Subject 6
Subject 7
Subject 8
Postcentral Sulcus
Precentral Sulcus
Inferior Frontal Sulcus
Superior Frontal Sulcus
Lateral Occip. S.

Figure S3. Preferred period of each recording site on the cortical surface for all subjects. Related to Figure 3C. Follows format of Figure S2.

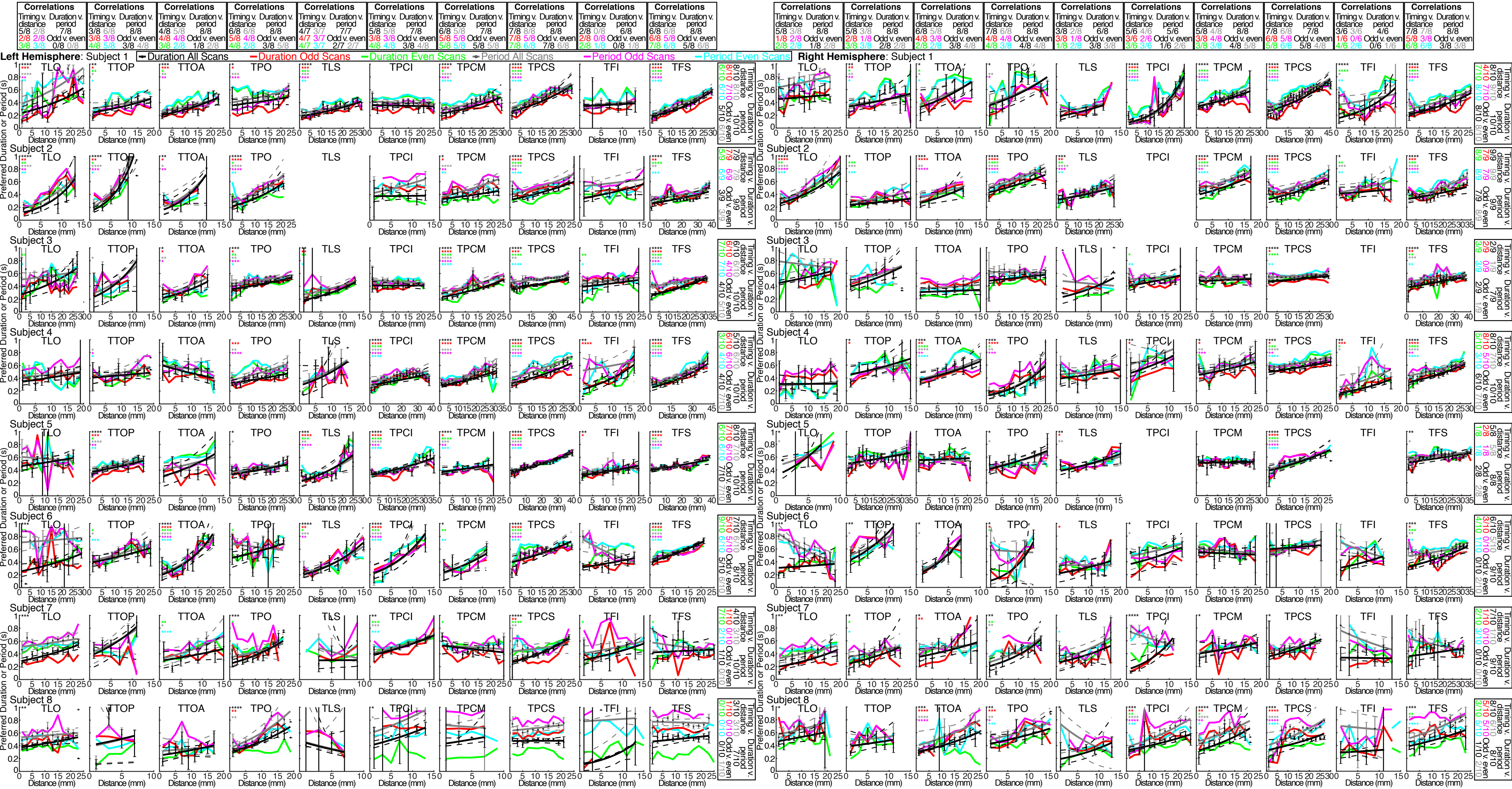

Left Hemisphere: Subject 1
Right Hemisphere: Subject 1
Duration All Scans
Duration Odd Scans
Duration Even Scans
Period All Scans
Period Odd Scans
Period Even Scans
Correlations
Timing v. distance
Duration v. period
Odd v. even
Subject 2
Subject 3
Subject 4
Subject 5
Subject 6
Subject 7
Subject 8
TLO
TTOP
TTOA
TPO
TLS
TPCI
TPCM
TPCS
TFI
TFS
Preferred Duration or Period (s)
Distance (mm)

Figure S4. Topographic progressions of preferred timing with distance across the timing maps. Related to Figure 4. Black and gray points show the mean preferred duration and period respectively in each bin of cortical distance, while error bars show the standard error of these means. Black and gray lines show the best fitting logarithmic functions to these points, with dashed lines showing the 95% confidence intervals of these fits determined by bootstrapping. Red and green lines join the mean preferred durations of each bin estimated from odd and even numbered scanning runs respectively. Cyan and magenta lines join the mean preferred periods of each bin estimated from odd and even numbered scanning runs respectively. Top boxes show for each map the count of individual subject's significant (p<0.05, FDR corrected) correlations of timing preferences with cortical surface distance (left), of duration preferences with period preferences (upper right), and of duration or period preferences estimated from odd and even runs (lower right). Middle and right boxes show for each hemisphere the count of individual maps showing significant correlations, following the same format. *p<0.05, **p<0.01, ***p<0.001, ****p<0.0001.

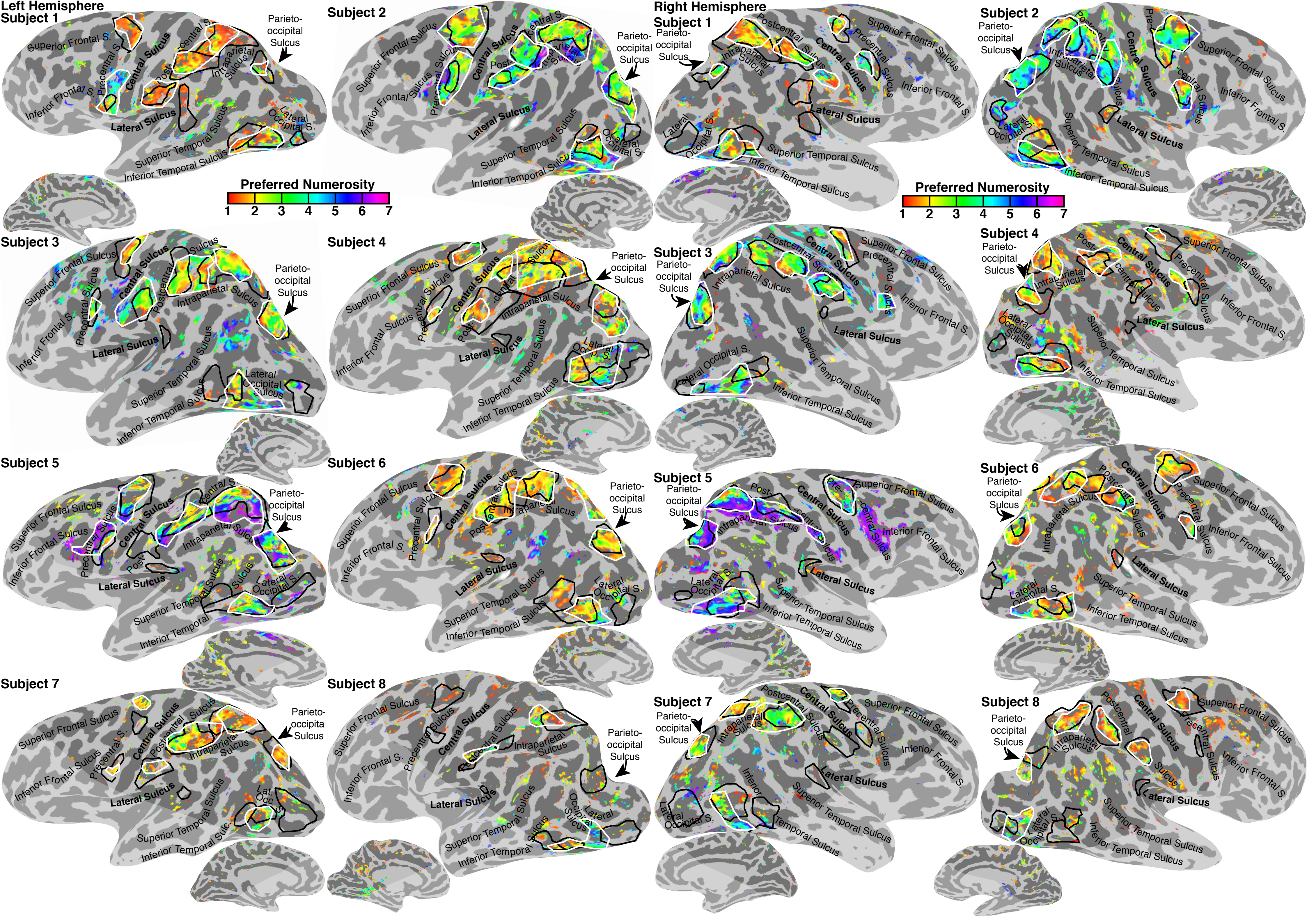

Left Hemisphere
Subject 1
Superior Frontal S.
Precentral S.
Central Sulcus
Postcentral S.
Intraparietal Sulcus
Parieto-occipital Sulcus
Inferior Frontal S.
Lateral Sulcus
Lateral Occipital S.
Superior Temporal Sulcus
Inferior Temporal Sulcus
Subject 2
Subject 3
Subject 4
Subject 5
Subject 6
Subject 7
Subject 8
Preferred Numerosity
1
2
3
4
5
6
7
Right Hemisphere
Subject 1
Postcentral Sulcus
Intraparietal Sulcus
Precentral Sulcus
Superior Frontal Sulcus
Inferior Frontal S.
Lateral Occipital S.
Subject 2
Subject 3
Subject 4
Subject 5
Subject 6
Subject 7
Subject 8
Preferred Numerosity
1
2
3
4
5
6
7

Figure S5. Preferred numerosity of each recording site on the cortical surface for all subjects. Related to Figure 7A. Follows format of Figure S2. Numerosity maps are outlined in white, while timing map locations are outlined in black. Recording sites with over 25% response variance explained by the response model are shown.

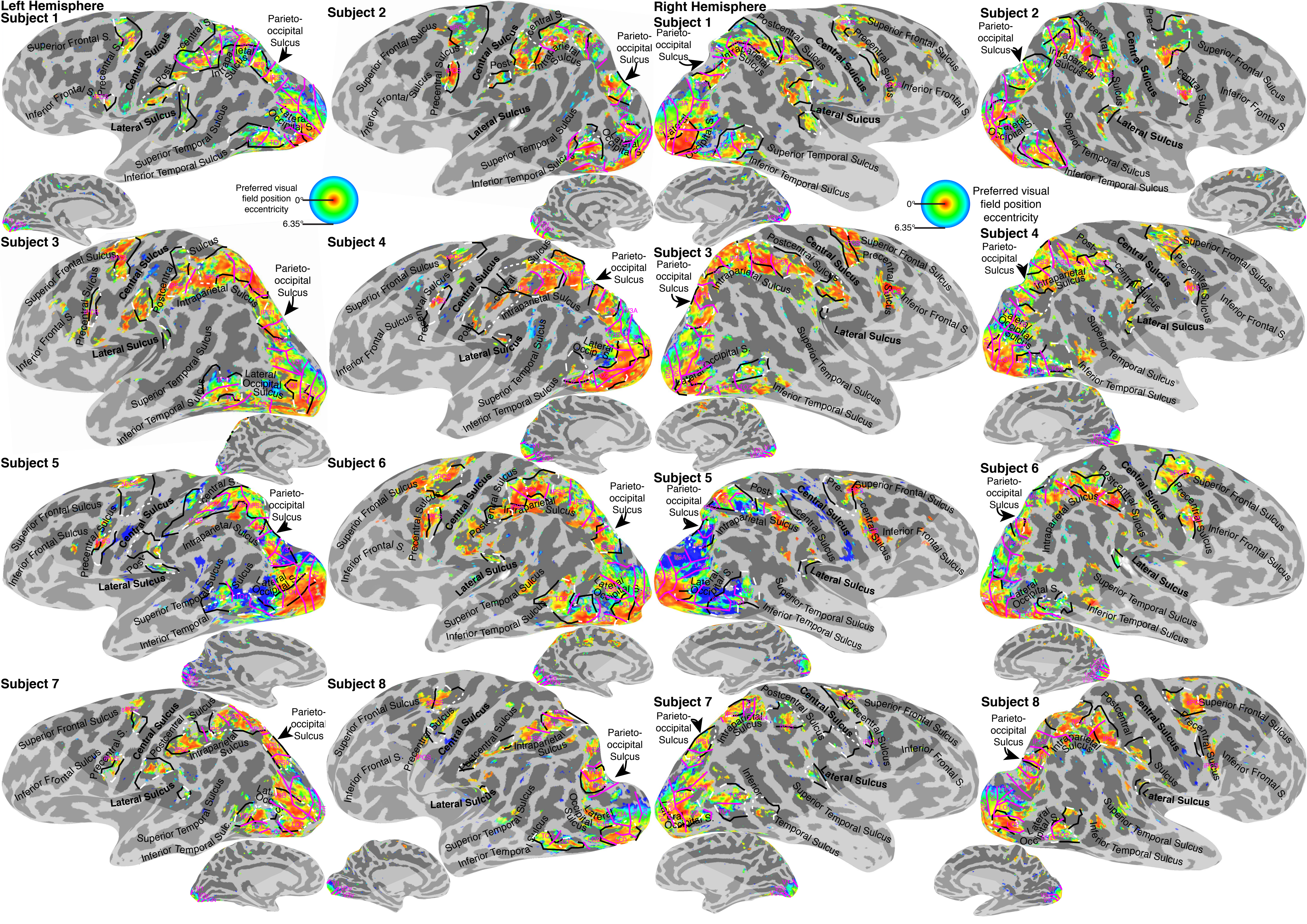

Left Hemisphere
Subject 1
Subject 2
Subject 3
Subject 4
Subject 5
Subject 6
Subject 7
Subject 8
Right Hemisphere
Subject 1
Subject 2
Subject 3
Subject 4
Subject 5
Subject 6
Subject 7
Subject 8
Preferred visual field position eccentricity
0°
6.35°
Parieto-occipital Sulcus
Superior Frontal Sulcus
Inferior Frontal Sulcus
Precentral Sulcus
Central Sulcus
Postcentral Sulcus
Intraparietal Sulcus
Lateral Sulcus
Lateral Occipital Sulcus
Superior Temporal Sulcus
Inferior Temporal Sulcus

Figure S6. Eccentricity of the preferred visual field positions of each recording site on the cortical surface for all subjects. Related to Figure 7B. Follows format of Figure S2. Visual field map borders shown as magenta lines, named in magenta text. Black and dashed white lines show timing map locations. Recording sites with over 10% response variance explained by the response model are shown.

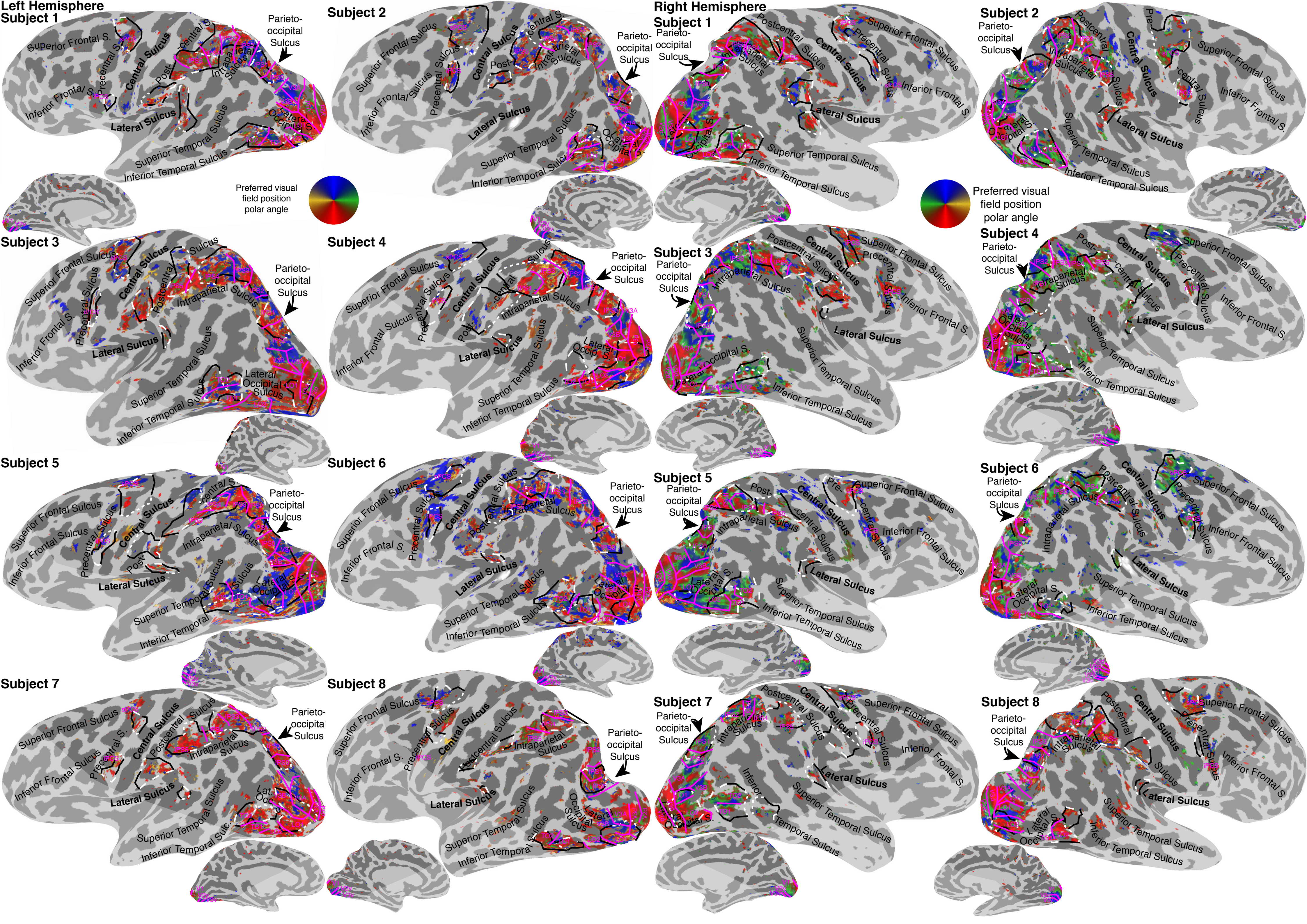

Left Hemisphere
Subject 1
Superior Frontal S.
Precentral S.
Central Sulcus
Post-
central S.
Intraparietal Sulcus
Parieto-occipital Sulcus
Inferior Frontal S.
Lateral Sulcus
Superior Temporal Sulcus
Inferior Temporal Sulcus
Lateral Occipital S.
Preferred visual field position polar angle
Subject 2
Superior Frontal Sulcus
Precentral Sulcus
Central Sulcus
Postcentral S.
Intraparietal Sulcus
Parieto-occipital Sulcus
Inferior Frontal Sulcus
Lateral Sulcus
Superior Temporal Sulcus
Inferior Temporal Sulcus
Lateral Occipital S.
Subject 3
Superior Frontal Sulcus
Precentral Sulcus
Central Sulcus
Postcentral Sulcus
Intraparietal Sulcus
Parieto-occipital Sulcus
Inferior Frontal S.
Lateral Sulcus
Superior Temporal Sulcus
Inferior Temporal Sulcus
Lateral Occipital Sulcus
Subject 4
Superior Frontal Sulcus
Precentral Sulcus
Central Sulcus
Intraparietal Sulcus
Parieto-occipital Sulcus
Inferior Frontal Sulcus
Lateral Sulcus
Superior Temporal Sulcus
Inferior Temporal Sulcus
Lateral Occip. S.
Subject 5
Superior Frontal Sulcus
Inferior Frontal Sulcus
Precentral Sulcus
Central Sulcus
Intraparietal Sulcus
Parieto-occipital Sulcus
Lateral Sulcus
Superior Temporal Sulcus
Inferior Temporal
Lateral Occipital S.
Subject 6
Superior Frontal Sulcus
Precentral Sulcus
Central Sulcus
Postcentral Sulcus
Intraparietal Sulcus
Parieto-occipital Sulcus
Inferior Frontal S.
Lateral Sulcus
Superior Temporal Sulcus
Inferior Temporal Sulcus
Lateral Occipital S.
Subject 7
Superior Frontal Sulcus
Precentral S.
Central Sulcus
Postcentral Sulcus
Intraparietal Sulcus
Parieto-occipital Sulcus
Inferior Frontal Sulcus
Lateral Sulcus
Superior Temporal Sulcus
Inferior Temporal Sulc.
Subject 8
Superior Frontal Sulcus
Precentral Sulcus
Central Sulcus
Postcentral Sulcus
Intraparietal Sulcus
Parieto-occipital Sulcus
Inferior Frontal S.
Lateral Sulcus
Superior Temporal Sulcus
Inferior Temporal
Lateral Occipital Sulcus
Right Hemisphere
Subject 1
Parieto-occipital Sulcus
Postcentral Sulcus
Intraparietal Sulcus
Central Sulcus
Precentral Sulcus
Superior Frontal Sulcus
Inferior Frontal S.
Lateral Sulcus
Lateral Occipital S.
Superior Temporal Sulcus
Inferior Temporal Sulcus
Subject 2
Parieto-occipital Sulcus
Postcentral Sulcus
Intraparietal Sulcus
Central Sulcus
Precentral Sulcus
Superior Frontal Sulcus
Inferior Frontal S.
Lateral Sulcus
Lateral Occipital S.
Superior Temporal Sulcus
Inferior Temporal Sulcus
Preferred visual field position polar angle
Subject 3
Parieto-occipital Sulcus
Postcentral Sulcus
Central Sulcus
Precentral Sulcus
Superior Frontal Sulcus
Intraparietal Sulcus
Inferior Frontal S.
Lateral Sulcus
Lateral Occipital S.
Superior Temporal Sulcus
Inferior Temporal Sulcus
Subject 4
Parieto-occipital Sulcus
Intraparietal Sulcus
Postcentral Sulcus
Central Sulcus
Precentral Sulcus
Superior Frontal Sulcus
Inferior Frontal S.
Lateral Sulcus
Lateral Occipital Sulcus
Superior Temporal Sulcus
Inferior Temporal Sulcus
Subject 5
Parieto-occipital Sulcus
Intraparietal Sulcus
Postcentral Sulcus
Central Sulcus
Precentral Sulcus
Superior Frontal Sulcus
Inferior Frontal Sulcus
Lateral Sulcus
Lateral Occipital S.
Superior Temporal Sulcus
Inferior Temporal Sulcus
Subject 6
Parieto-occipital Sulcus
Intraparietal Sulcus
Postcentral Sulcus
Central Sulcus
Precentral Sulcus
Superior Frontal Sulcus
Inferior Frontal Sulcus
Lateral Sulcus
Lateral Occipital S.
Superior Temporal Sulcus
Inferior Temporal Sulcus
Subject 7
Parieto-occipital Sulcus
Postcentral Sulcus
Intraparietal Sulcus
Central Sulcus
Precentral Sulcus
Superior Frontal Sulcus
Inferior Frontal S.
Lateral Sulcus
Lateral Occipital S.
Superior Temporal Sulcus
Subject 8
Parieto-occipital Sulcus
Postcentral
Intraparietal Sulcus
Central Sulcus
Precentral Sulcus
Superior Frontal Sulcus
Inferior Frontal S.
Lateral Sulcus
Lateral Occipital S.
Superior Temporal Sulcus
Inferior Temporal Sulcus

Figure S7. Polar angle of the preferred visual field positions of each recording site on the cortical surface for all subjects. Related to Figure 7C. Follows format of Figure S6.

	Volumetric transformation<br>Mean (sd) center MNI x,y,z coordinates		Surface-based transformation<br>Mean (sd) center Talairach N27 x,y,z coordinates	
Map	Left hemisphere	Right hemisphere	Left hemisphere	Right hemisphere
TLO	-30(5), -91(4), -7(7)	27(6), -91(5), -1(7)	-26(5), -81(4), 6(8)	26(7), -82(3), 6(7)
TTOP	-41(7), -76(7), -4(7)	45(5), -71(4), -3(7)	-40(5), -66(4), 6(6)	42(4), -61(2), 4(5)
TTOA	-46(3), -64(6), -1(5)	47(4), -61(4), -2(3)	-44(3), -55(4), 8(4)	45(2), -52(3), 6(4)
TPO	-25(3), -79(7), 26(6)	29(4), -74(6), 28(6)	-21(3), -70(7), 34(3)	26(4), -65(4), 35(3)
TLS	-48(6), -37(5), 20(4)	47(8), -36(4), 18(6)	-47(4), -32(6), 22(4)	46(6), -29(3), 23(6)
TPCI	-51(7), -29(4), 34(5)	52(11), -31(8), 37(5)	-47(5), -20(4), 37(4)	47(9), -26(8), 39(2)
TPCM	-36(4), -43(6), 45(9)	34(6), -43(5), 46(8)	-34(2), -35(4), 49(8)	29(6), -39(4), 49(4)
TPCS	-23(6), -63(7), 55(7)	23(7), -64(8), 54(5)	-21(6), -56(4), 56(4)	18(6), -58(4), 57(4)
TFI	-55(3), -2(6), 36(3)	51(4), -1(4), 35(5)	-51(2), 2(3), 41(3)	49(2), -1(3), 37(7)
TFS	-24(4), -14(5), 50(5)	28(4), -13(5), 51(4)	-24(4), -8(5), 52(3)	26(3), -10(4), 54(3)

Table S1. Distribution of coordinates of each map's center across subjects. Related to Figure 3A. In Montreal Neurological Institute (MNI) and Talairach N27 template spaces, following volumetric and surface-based transformations respectively.
